# A Naphthalimide‐Based NIR‐Emissive AIE Photosensitizer for Synergistic Apoptosis/Ferroptosis‐Mediated Antitumor Therapy and Broad‐Spectrum Bacterial Inactivation With Rapid Wound Healing

**DOI:** 10.1002/adhm.71493

**Published:** 2026-07-29

**Authors:** Sayed Mir Sayed, Zeeshan Tahir, Fahri Alkan, Yavuz Nuri Ertas

**Affiliations:** ^1^ ERNAM–Nanotechnology Research and Application Center Erciyes University Kayseri Türkiye; ^2^ Department of Medical Biochemistry Faculty of Medicine Erciyes University Kayseri Türkiye; ^3^ Department of Chemistry Bilkent University Ankara Türkiye; ^4^ Department of Biomedical Engineering Erciyes University Kayseri Türkiye

**Keywords:** aggregation‐induced emission, antitumor photodynamic therapy, apoptosis, bacterial imaging, ferroptosis, plasma membrane staining, reactive oxygen species, wound healing

## Abstract

Fluorescence‐guided photodynamic therapy (PDT) that targets both malignant cells and pathogenic bacteria is attractive yet challenging, because a single photosensitizer (PS) must combine near‐infrared (NIR) emission, efficient reactive oxygen species (ROS) generation, and strong membrane affinity across distinct biological envelopes. Here, we report a donor–*π*‐bridge‐engineered series of ionic naphthalimide luminogens, TPA‐NIM‐M, TPAV‐NIM‐M, and TPAPV‐NIM‐M, featuring aggregation‐induced emission (AIE) and a cationic membrane‐anchoring motif. Among them, TPAPV‐NIM‐M exhibits far‐red/NIR emission, high photostability, and enhanced ROS generation under white‐light irradiation through both type I and type II pathways. The extended donor–*π* conjugation promotes photosensitization, while the ionic amphiphilic structure enables stable plasma membrane (PM) localization, strong membrane binding, and prolonged membrane retention. In cancer cells, photoactivation of TPAPV‐NIM‐M induces oxidative membrane damage and regulated cell death involving apoptosis and lipid‐peroxidation‐associated ferroptosis, while allowing real‐time fluorescence tracking of membrane blebbing during apoptosis. TPAPV‐NIM‐M also stains both Gram‐negative and Gram‐positive bacteria and enables efficient photodynamic bacterial inactivation. In vivo, TPAPV‐NIM‐M promotes healing in an *Escherichia coli*‐infected wound model and suppresses tumor growth under local light irradiation without apparent systemic toxicity. This work establishes ionic naphthalimide AIEgens as a powerful class of membrane‐anchored theranostic agents for image‐guided antitumor and antibacterial PDT with broad biomedical potential and translational promise.

## Graphical abstract

An ionic naphthalimide AIE photosensitizer (PS), TPAPV‐NIM‐M, anchors to cellular and bacterial membranes, emits in the far‐red/near‐infrared (NIR) region, and efficiently generates reactive oxygen species (ROS) under white light. Its membrane‐localized photodynamic action drives apoptosis/ferroptosis‐mediated tumor suppression and broad‐spectrum bacterial inactivation, enabling image‐guided anticancer therapy and accelerated healing of infected wounds.

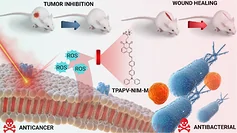

## 1 Introduction

Cancer remains a major global health threat associated with high morbidity and mortality. According to the World Health Organization (WHO), 20 million new cancer cases and 9.7 million deaths were reported in 2022. In parallel, with the emergence of drug‐resistant bacterial strains, infections caused by pathogenic bacteria are among the leading causes of high mortality worldwide, and it is projected that over the next 25 years, 29 million people will be affected by such infections [1]. Radiation therapy, surgery, and chemotherapy are commonly used methods for cancer treatment; however, they have several disadvantages, such as invasiveness, drug resistance, complicated procedures, and severe side effects [2, 3, 4, 5, 6, 7]. In addition, antibiotics play a crucial role in combating bacterial infections; however, their overuse accelerates pathogen evolution, leading to drug resistance among bacterial strains [8]. In this regard, photodynamic therapy (PDT), with its low toxicity, high spatiotemporal selectivity, low likelihood of inducing resistance, noninvasiveness, controllability, and ease of operation, is highly desirable in oncology and the treatment of infectious diseases caused by drug‐resistant pathogens [9, 10, 11, 12, 13]. PDT is an effective therapeutic modality that relies on a photosensitizer (PS), light, and endogenous oxygen. To carry out effective photodynamic ablation of cancer and pathogenic bacteria, a PS is first activated by a specific wavelength of light, generating reactive oxygen species (ROS) that induce oxidative damage to biological molecules, such as proteins, lipids, and DNA, present in the target cells [14]. An ideal PS should possess excellent brightness in the aggregated state, negligible dark toxicity, effective light absorption, and high ROS production. Conventional PSs, such as cyanines, phthalocyanines, porphyrins, quantum dots, transition metal complexes, and phenothiazine derivatives, are commonly used in photoinduced anticancer applications [15, 16, 17, 18, 19, 20]. However, these PSs suffer from loss of fluorescence signals in aggregated state due to aggregation‐caused quenching (ACQ), insufficient ROS generation, and low photostability, which greatly limit their practical clinical applications [21]. Therefore, there is an urgent need to develop novel PSs with high ROS‐generating capacity, excellent brightness in the aggregated state, high photostability, and biocompatibility.

To overcome ACQ, a new family of organic molecules with aggregation‐induced emission (AIE) properties was reported in 2001, offering a better alternative to conventional PSs. AIE luminogens (AIEgens) are usually designed with nonplanar structures to resist *π*–*π* stacking interactions [22] and are nonfluorescent or weakly fluorescent in solution, but emit bright fluorescence upon aggregation, which is attributed to the restriction of intramolecular motion [23, 24]. Due to their high photostability, biocompatibility, and bright fluorescence in the aggregated state, AIEgens are used in diverse biomedical applications, including bioimaging and biosensing [25, 26, 27, 28, 29, 30]. More importantly, in the aggregated state, AIEgens block nonradiative pathways, leading to high quantum yield and stabilization of the triplet excited state, thereby facilitating ROS generation for the photodynamic ablation of cancer cells and bacteria [31, 32, 33]. In addition, their structures can be easily tuned to target the desired cell organelles with high accuracy and are suitable candidates for targeted PDT, which is advantageous over conventional PDT, as the lifetime of intracellular ROS produced by PSs is about 200 ns with a diffusion distance of 0.01–0.1 µm, meaning that the ROS could be consumed before reaching the desired target, which significantly affects the therapeutic efficacy [14, 34, 35]. Targeted PDT is therefore highly desirable because it enables ROS generation in close proximity to the intended biological target [36, 37, 38, 39, 40].

The plasma membrane (PM) is the cell's protective envelope that maintains its integrity and separates the cell's interior from the external environment [41]. The PM is mainly composed of phospholipids, which contain hydrophilic head groups and hydrophobic hydrocarbon tails, as well as proteins and carbohydrates [42]. The PM plays a key role in various cellular processes, including signal transduction, cell proliferation and migration, exocytosis and endocytosis, substance exchange and uptake, and intercellular communication. In addition, PM morphology provides valuable information about cell health, as many cellular processes, such as necrosis, apoptosis, and pyroptosis, deform or rupture the PM, leading to the leakage of intracellular contents and ultimately causing irreversible cell death [42, 43]. Moreover, the PM is considered an attractive target for PDT because therapeutic agents can act at the cell surface without crossing the membrane, thereby circumventing the poor membrane permeability of negatively charged molecules and antibody‐based therapeutics [44]. PMs can also provide an extensive, easily targeted surface area, reducing off‐target effects. PMs of normal and cancer cells differ in specific receptors and membrane potential, which help in the selective targeting of tumors by therapeutic agents. PM‐targeted PSs do not need to cross the PM barrier. Instead, light activation at the PM generates ROS locally, damaging the PM and promoting cell death. However, it is challenging to synthesize PSs with specific PM targeting ability and prolonged retention at the PM, as small‐molecule PSs can easily diffuse through the PM to the cytoplasm, which decreases the efficacy of PDT against cancer cells. Similarly, bacterial cell membranes are composed of amphiphilic lipids and are negatively charged, which could make them ideal targets for an amphiphilic PS to establish strong electrostatic interactions with these surfaces, thereby facilitating PS insertion into the lipid membrane [45]. Under light irradiation, PSs are activated to generate ROS, inducing irreversible damage to the cell membrane and DNA, making it harder for pathogens to resist photodynamic inactivation even after mutation, as PDT acts on multiple targets and is less likely to induce pathogen resistance [46]. Effective photodynamic elimination of pathogenic bacteria requires a PS with an appropriate balance of hydrophilicity and hydrophobicity. The PS must remain associated with the bacterial surface while also penetrating the membrane and establishing electrostatic and hydrophobic interactions. Excessive hydrophilicity may favor surface binding but limit membrane penetration, thereby reducing photodynamic antibacterial efficacy [47].

**SCHEME 1:**
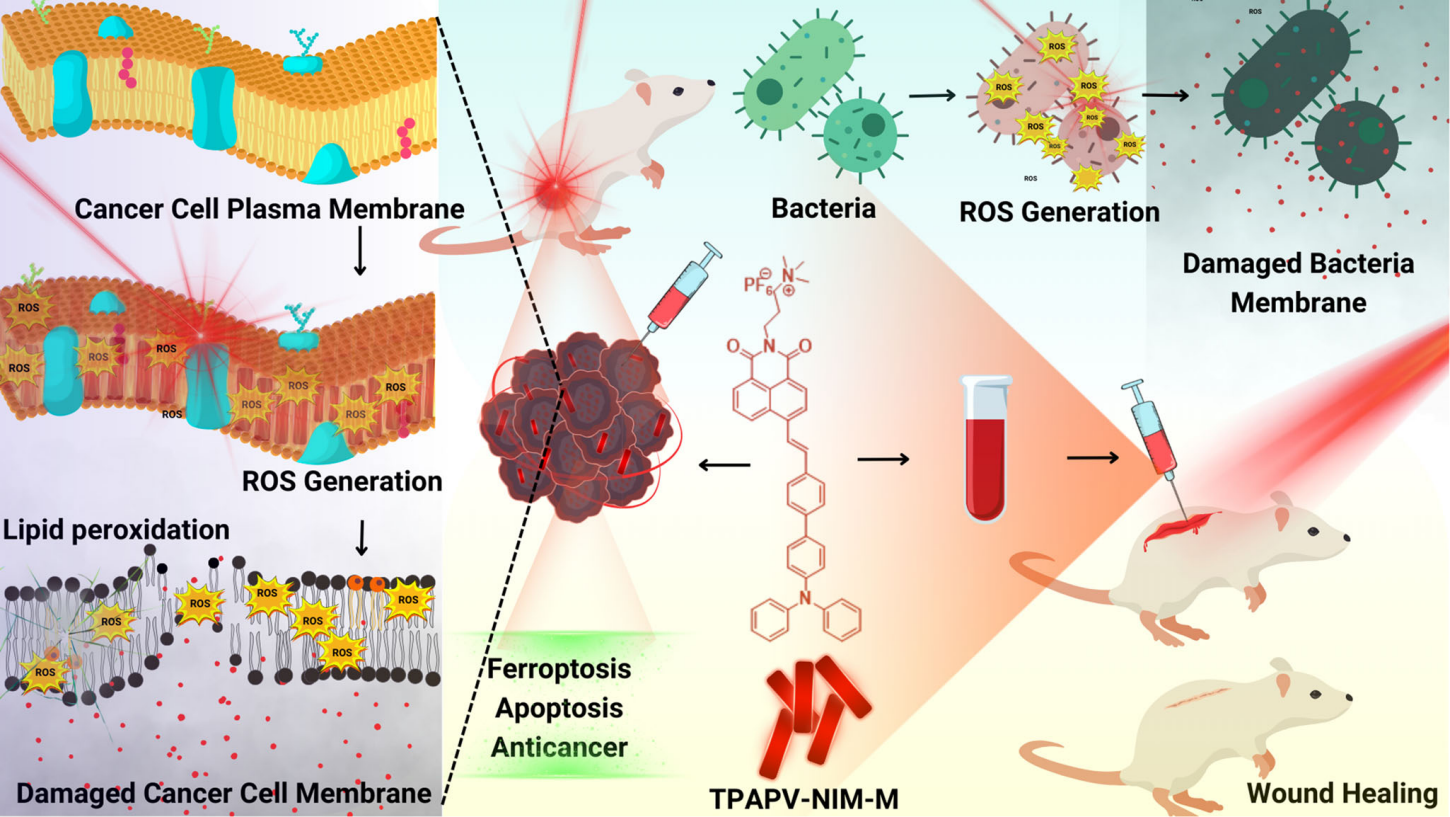
Schematic illustration of TPAPV‐NIM‐M‐mediated membrane‐targeted PDT for cancer cells and bacteria, including ROS generation, cancer‐cell membrane damage, apoptosis/ferroptosis‐associated cell death, bacterial membrane disruption, and wound healing in an infected wound model.

Inspired by the effectiveness of PDT in antitumor and antibacterial applications, herein, we report the synthesis and application of three novel naphthalimide‐based donor–*π*–acceptor (D–*π*–A) type near‐infrared (NIR)–emitting AIE PSs, TPA‐NIM‐M, TPAV‐NIM‐M, and TPAPV‐NIM‐M. Although naphthalimide‐based fluorescent molecules have been extensively studied for biosensing applications, they have rarely been explored for photosensitizing applications [48, 49, 50, 51]. We present a novel *π*‐bridge‐ and donor‐engineering strategy to enhance the photosensitization of naphthalimide‐based fluorescent molecules, in which *π*‐bridging and donor modulation markedly improve ROS generation. TPAPV‐NIM‐M, with extended *π*‐conjugation, is an NIR‐emissive, photostable, biocompatible, and AIE‐active PS that exhibits superior ROS (type I and type II) generation and was therefore evaluated for PDT of cancer cells and pathogens. It shows strong binding affinity to the PM of mammalian cells and can also stain both Gram‐negative (G^−^) and Gram‐positive (G^+^) bacteria. Compared with TPA‐NIM‐M, TPAV‐NIM‐M, and commercial PSs, such as Rose bengal (RB) and chlorin e6 (Ce6), TPAPV‐NIM‐M exhibits higher ROS generation under white‐light irradiation and demonstrates excellent performance in photodynamic ablation of cancer and bacterial cells (Scheme 1).

## 2 Results and Discussion

### 2.1 Molecular Design and Photophysical Properties

Incorporating the push–pull effect into an organic molecule is a primary strategy for achieving longer‐wavelength absorption and emission, features that are essential for its application as a PS. To achieve a push–pull effect, donor and acceptor units are typically incorporated into the molecular structure to facilitate intramolecular charge transfer (ICT) and enhance ROS generation [52]. A strong D–A interaction spatially separates the electron densities of the highest occupied molecular orbital (HOMO) and the lowest unoccupied molecular orbital (LUMO), thereby reducing the energy gap (∆*E*_ST_) between the singlet (S_1_) and triplet (T_1_) states and facilitating intersystem crossing (ISC), a necessary condition for ROS generation [53, 54, 55]. Following the D–A strategy, three novel PSs, TPA‐NIM‐M, TPAV‐NIM‐M, and TPAPV‐NIM‐M, were synthesized via Suzuki and Heck cross‐coupling reactions in which naphthalimide was used as an electron‐accepting core and triphenylamine (TPA) as an electron‐donor moiety. To achieve longer‐wavelength absorption and emission and to induce a strong ICT effect in the designed molecules, the donor and acceptor moieties were separated via a *π*‐bridge in TPAV‐NIM‐M and TPAPV‐NIM‐M (Figure 2a). To promote effective cellular interactions, a quaternary ammonium unit was incorporated into the acceptor moiety as an electron‐deficient cation, which can also facilitate charge transfer from the electron‐donor moiety (TPA) to the naphthalimide acceptor, thereby inducing a strong D–A effect in the molecules [56]. In addition to its electron‐donating ability, TPA, with a twisted molecular structure, also introduces steric hindrance, thereby minimizing fluorescence quenching due to *π*–*π* stacking in planar aromatic structures [14].

**FIGURE 1:**
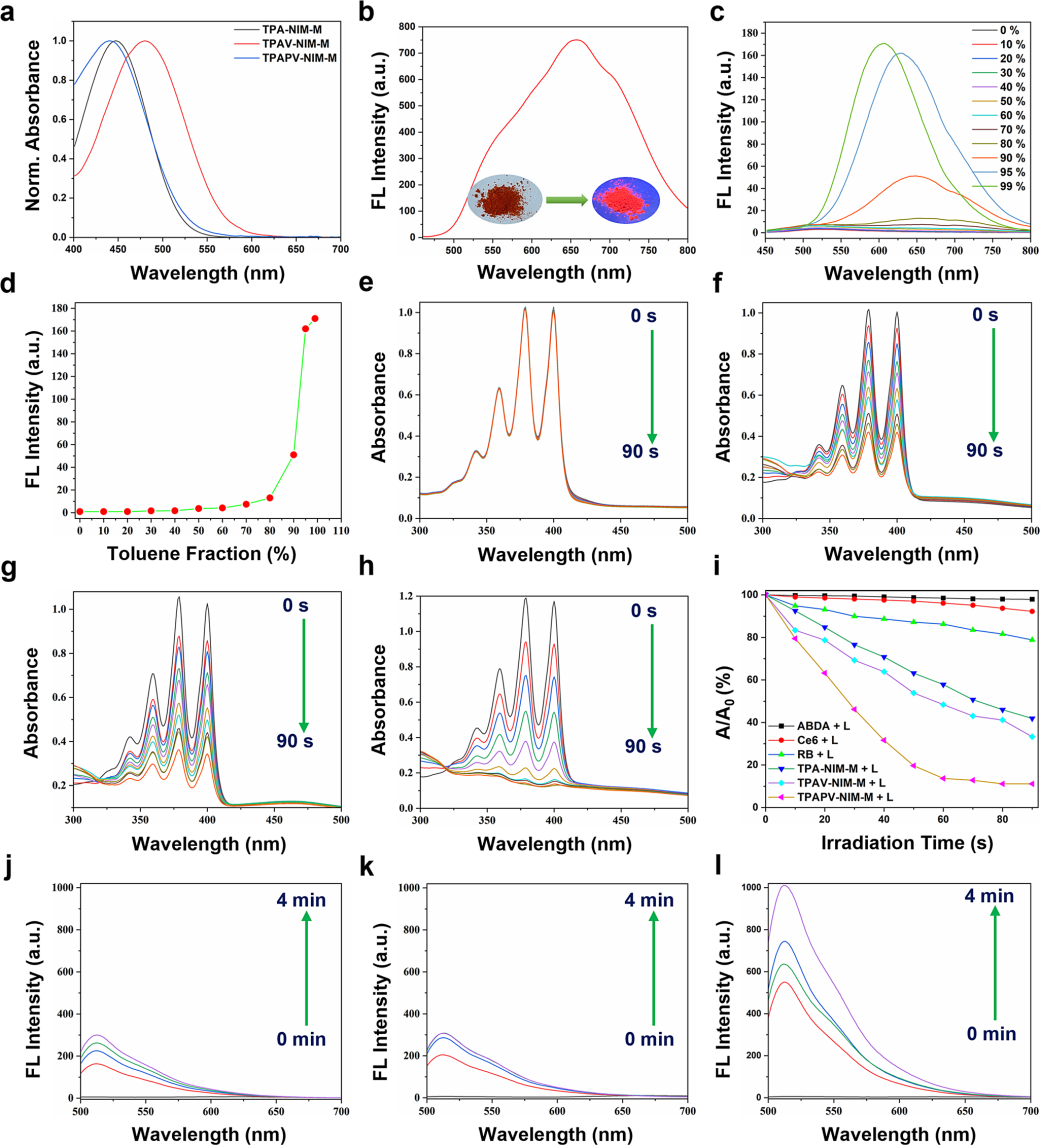
(a) Absorption spectra of TPA‐NIM‐M, TPAV‐NIM‐M, and TPAPV‐NIM‐M in chloroform. (b) Fluorescence emission spectrum of TPAPV‐NIM‐M in chloroform. (Inset: Photographs of TPAPV‐NIM‐M powder in daylight and under a 365 nm UV lamp). (c) Fluorescence emission spectrum of TPAPV‐NIM‐M in DMSO/toluene mixtures with different volume fractions of toluene. (d) Plot of the changes in the fluorescence intensities of TPAPV‐NIM‐M with varying fractions of toluene. (e) Absorption spectra of ABDA (75 µM) under white‐light irradiation (∼27 mW/cm^2^). (f–h) Absorption spectra of ABDA (75 µM) in the presence of TPA‐NIM‐M (5 µM), TPAV‐NIM‐M (5 µM), and TPAPV‐NIM‐M (5 µM) under white‐light irradiation (∼27 mW/cm^2^). (i) Degradation rates of ABDA in the presence of different PSs (TPA‐NIM‐M, TPAV‐NIM‐M, TPAPV‐NIM‐M, Ce6, and RB), where *A*/*A*_0_ represents the absorbance of ABDA at 400 nm. *Note*: L in (i) stands for light. (j–l) Changes in the fluorescence emission spectra of HPF (5 µM) under white‐light irradiation (∼27 mW/cm^2^) in the presence of TPA‐NIM‐M (5 µM), TPAV‐NIM‐M (5 µM), and TPAPV‐NIM‐M (5 µM).

The photophysical properties of the synthesized fluorophores were investigated using UV–vis absorption and fluorescence spectroscopy (Figure 1). In chloroform, the absorption maxima of TPA‐NIM‐M, TPAV‐NIM‐M, and TPAPV‐NIM‐M were observed at 448, 480, and 442 nm, respectively. It was expected that TPAPV‐NIM‐M, with extended *π*‐conjugation, would exhibit a red‐shifted absorption maximum. However, it showed a slight blue shift, which may be attributed to its reduced molecular planarity compared with TPA‐NIM‐M and TPAV‐NIM‐M [1]. The molar absorption coefficients (*ɛ*) of the synthesized AIEgens were also measured in dimethyl sulfoxide (DMSO) (Figure S1). TPAPV‐NIM‐M exhibited the highest *ɛ* (26 095 M^−1^ cm^−1^) at its absorption maximum, compared to TPA‐NIM‐M (6435 M^−1^ cm^−1^) and TPAV‐NIM‐M (19 490 M^−1^ cm^−1^). This enhancement may be attributed to the strong D–A characteristics and extended *π*‐conjugation of TPAPV‐NIM‐M, which endow it with strong light‐harvesting ability [57, 58]. The emission maxima for TPA‐NIM‐M, TPAV‐NIM‐M, and TPAPV‐NIM‐M were observed at 628 (Figure S2a), 657 (Figure S2d), and 656 nm (Figure 1b), with Stokes shifts calculated to be 180, 177, and 214 nm, respectively, which are large enough to avoid the self‐quenching of the synthesized AIEgens in the aggregated state [59]. These AIEgens, bearing a TPA moiety, were expected to exhibit AIE behavior; to confirm our hypothesis, DMSO/toluene mixtures were used. In pure DMSO, the synthesized AIEgens exhibited weak or no fluorescence, which is attributed to the twisted ICT (TICT) effect in a polar medium (Figure 1c,d and Figure S2b,e) [60], while in DMSO/toluene mixtures, with an increase in the toluene fraction, fluorescence increased and reached the maximum at 95%, 90%, and 99% toluene fractions for TPA‐NIM‐M, TPAV‐NIM‐M, and TPAPV‐NIM‐M, respectively. A slight blue shift was also observed as the polarity of the solvent mixture decreased, consistent with TICT behavior [61].

### 2.2 Light‐Triggered ROS Generation

To evaluate the practicality of the synthesized fluorophores, the ROS‐generating ability of the synthesized AIEgens was assessed using anthracenediyl‐bis‐(methylene)‐dimalonic acid (ABDA), hydroxyphenyl fluorescein (HPF), and dihydrorhodamine 123 (DHR‐123), which are commercial sensors for the detection of singlet oxygen (^1^O_2_), hydroxyl radicals (OH^•^), and superoxide anions (O_2_^•–^), respectively. These AIEgens exhibit strong absorption in the visible region (Figure 1a); therefore, readily available white light was used to generate ROS in an aqueous medium with ABDA at 75 µM and each PS at 5 µM. Before the experiment, the photostability of ABDA (75 µM) was assessed under white‐light irradiation (∼27 mW/cm^2^), and no ABDA decomposition was observed after 90 s of irradiation (Figure 1e). Next, the decomposition of ABDA in the presence of TPA‐NIM‐M, TPAV‐NIM‐M, and TPAPV‐NIM‐M was studied under white‐light irradiation (∼27 mW/cm^2^) for 0–90 s, and Figures 1f–h show that TPA‐NIM‐M, TPAV‐NIM‐M, and TPAPV‐NIM‐M consumed 58%, 67%, and 90% of ABDA, respectively. For comparison, two commercial PSs, Ce6 and RB, were used under the same conditions as the synthesized PSs, and it was found that Ce6 consumed 9% of ABDA (Figure S3a), while RB consumed up to 20% of ABDA (Figure S3b). The comparative ABDA degradation rates in the presence of TPA‐NIM‐M, TPAV‐NIM‐M, TPAPV‐NIM‐M, Ce6, and RB are depicted in Figure 1i. These results indicate that TPAPV‐NIM‐M exhibits a superior ROS‐generating ability compared to TPA‐NIM‐M, TPAV‐NIM‐M, and the commonly used commercial PSs Ce6 and RB. Similarly, the generation of OH^•^ and O_2_^•−^ was also assessed in an aqueous medium by separately mixing 5 µM of TPA‐NIM‐M, TPAV‐NIM‐M, and TPAPV‐NIM‐M with HPF (10 µM) and DHR‐123 (5 µM) in a 1:1 ratio, respectively, followed by white‐light irradiation (∼27 mW/cm^2^). Prolonged irradiation enhanced ROS generation, as evidenced by higher fluorescence intensities at 515 nm (Figure 1j–l) and 527 nm (Figure S4). The fluorescence intensities of HPF and DHR‐123 treated with TPAPV‐NIM‐M were higher than those of TPA‐NIM‐M and TPAV‐NIM‐M, indicating that TPAPV‐NIM‐M has a superior ROS‐generating ability (type I and type II) under white‐light irradiation compared to TPA‐NIM‐M and TPAV‐NIM‐M, and is a suitable candidate for PDT via both type I and type II pathways.

**FIGURE 2:**
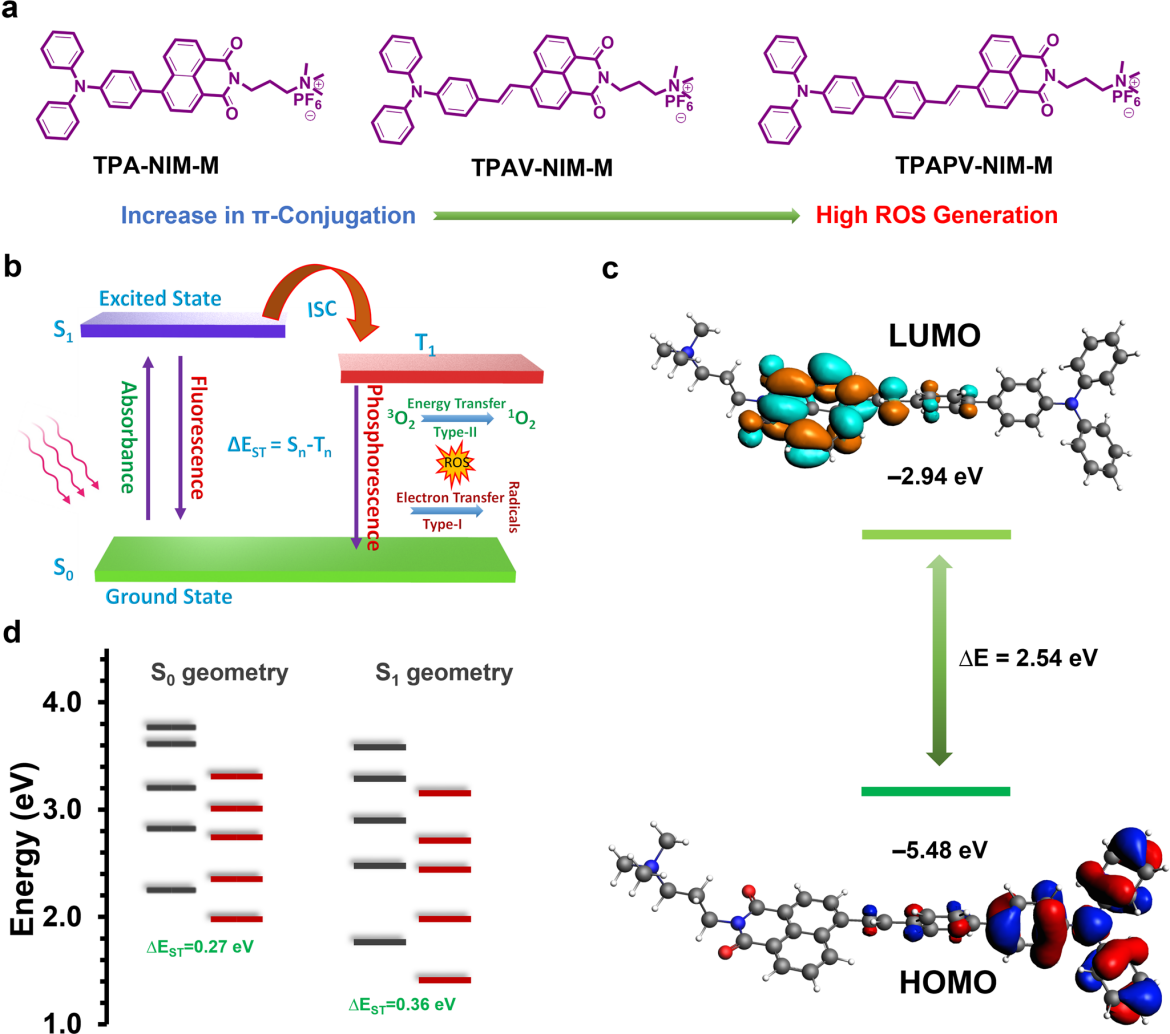
(a) Molecular structures of TPA‐NIM‐M, TPAV‐NIM‐M, and TPAPV‐NIM‐M. (b) Schematic illustration of the ROS generation via type I and type II pathways. (c) Pictorial representations of HOMO and LUMO energy levels of TPAPV‐NIM‐M. (d) Singlet and triplet energy levels of TPAPV‐NIM‐M calculated with TDDFT using ground‐state geometry and B3LYP/DZP level of theory.

To further understand the ROS generation mechanism and photophysical properties of TPAPV‐NIM‐M, density functional theory (DFT) and time‐dependent DFT (TD‐DFT) calculations were performed at the B3LYP/DZP level of theory (see Supporting Information for computational details). The calculated UV–vis absorption spectra of TPAPV‐NIM‐M are presented in Figure S5, while Tables S1–S4 summarize the excited‐state properties at both S_0_ and S_1_ optimized geometries in chloroform and water. The theoretical absorption spectra reproduce the main experimental features (Figure 1a,b), and the calculated Stokes shifts agree well with the measured values. In all cases, the lowest‐lying singlet excited state (S_1_) is predominantly characterized by a HOMO→LUMO (97%) transition. The HOMO and LUMO energies were calculated to be −5.48 and −2.94 eV, respectively, and the HOMO–LUMO gap was found to be 2.54 eV. Meanwhile, T_1_ exhibits a mixed character, with the HOMO→LUMO (49%–52%) transition showing the largest coefficient. The pictorial representation of the frontier molecular orbitals (Figure 2c) and the calculated oscillator strength suggests that S_1_ exhibits a hybridized local‐ and charge‐transfer (HLCT) character [62, 63]. In addition, the calculated Λ (lambda) index [64], which quantifies the spatial overlap between the hole and electron densities for the excited‐state wavefunction, indicates that S_1_ is a CT‐dominated HLCT state. A similar HLCT character is observed for the T_1_ state; however, the charge‐transfer contribution is somewhat reduced in T_1_ because of greater configurational mixing.

ROS generation requires population of the triplet excited state via intersystem crossing (ISC) from initially excited singlet states [65]. According to Fermi's golden rule, the ISC rate can be expressed as

$$k_{ISC}=\frac{2\pi }{\hbar }{\left|\langle T_{n}\left|{\hat{H}}_{SOC}\right|S_{m}\rangle \right|}^{2}\rho_{FC}$$

This expression indicates that ISC efficiency is governed by two key factors: the magnitude of the spin‐orbit coupling (SOC) matrix elements and the energy difference between the singlet and triplet states (Δ*E*_ST_). TPAPV‐NIM‐M, with strong CT character, has calculated ∆*E*_ST_ values of 0.27 and 0.36 eV for S_0_ and S_1_ geometries, respectively, indicating a moderately small energy gap to facilitate S_1_→T_1_ population transfer (Figure 2d). Meanwhile, the SOC matrix elements are calculated to be nonzero (0.36 and 0.21 cm^−1^) due to different degrees of CT character of S_1_ and T_1_ states (Tables S5 and S6). In addition to the direct S_1_→T_1_ pathway, higher‐lying triplet states may be involved in the ISC process via a hot‐exciton mechanism, as the ∆*E*_ST_ and SOC between the S_1_ and T_2_ states indicate a favorable pathway for population transfer [63, 66]. These results suggest that the combined effects of SOC, ∆*E*_ST_, possible spin–vibronic coupling/effects [67], and hot‐exciton‐mediated S_1_→T_n_ channels may support efficient triplet formation for TPAPV‐NIM‐M, making it a promising PS for ROS generation. TPAPV‐NIM‐M, which generates high levels of ROS (types I and II) in an aqueous medium, was further evaluated for PDT of cancer cells and pathogenic bacteria.

### 2.3 Confocal Imaging Study

Prior to cell imaging, we evaluated the cytotoxicity of the synthesized TPAPV‐NIM‐M in MDA‐MB‐231 and HUVEC cells because cytotoxicity is an essential parameter for assessing the biosafety of molecules intended for biomedical applications. To evaluate the dark cytotoxicity, MDA‐MB‐231 and HUVEC cells were incubated with different concentrations (0–40 µM) of TPAPV‐NIM‐M for 24 h in the dark. Thereafter, cell viability was determined using the standard MTT assay. The cell viability of the HUVECs was 100% at 20 µM and decreased to 73% at 40 µM, whereas the cell viability of the MDA‐MB‐231 cells was 100% at 30 µM and decreased to 85% at 40 µM (Figure S6a,b). These results demonstrate that TPAPV‐NIM‐M is safe for biomedical applications with negligible dark toxicity at 20 or 30 µM, although it induced slight cytotoxicity at a higher concentration (40 µM). Given its strong absorption in the visible region, intense emission in the NIR‐I region, high ROS generation in an aqueous medium, and low dark cytotoxicity, we further investigated the cellular interactions of TPAPV‐NIM‐M via confocal laser scanning microscopy, as fluorescence imaging techniques play a key role in the visualization of cellular and subcellular structures without any considerable damage to cells.

**FIGURE 3:**
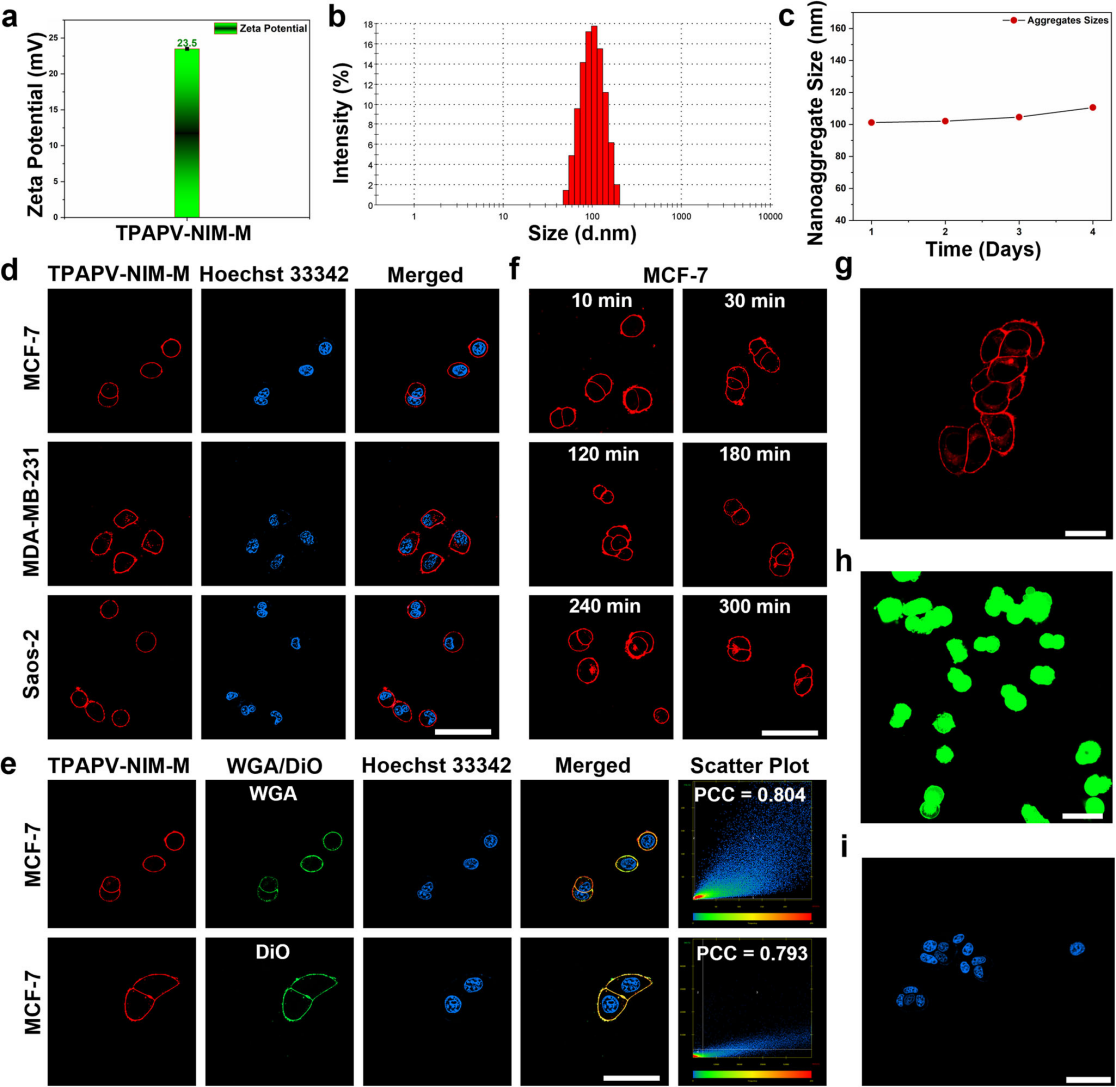
(a) Zeta potential of TPAPV‐NIM‐M in water. (b) Size distribution of nanoaggregates of TPAPV‐NIM‐M (5 µM) in PBS. (c) Changes in the sizes of TPAPV‐NIM‐M (5 µM) nanoaggregates in PBS (days 1–4). (d) Confocal fluorescence images of MCF‐7, MDA‐MB‐231, and Saos‐2 cells incubated with TPAPV‐NIM‐M (5 µM) and Hoechst 33342 (5 µM). (e) Confocal fluorescence images of MCF‐7 cells costained with TPAPV‐NIM‐M (5 µM), WGA‐conjugates (5 µM), DiO (10 µM), and Hoechst 33342 (5 µM). (f) Time‐dependent internalization (from 10–300 min) of TPAPV‐NIM‐M (5 µM) in MCF‐7 cells. TPAPV‐NIM‐M was excited with a 488 nm laser, and the emission was collected between 610 and 700 nm. Hoechst 33342 was excited by a 405 nm laser, and emission was collected between 400 and 515 nm. WGA‐conjugates and DiO were excited with a 488 nm laser, and their emissions were collected between 450 and 560 nm. Scale bar: 10 µm. (g) Confocal microscope image of MCF‐7 cells treated with TPAPV‐NIM‐M (5 µM) for 24 h. Scale bar: 10 µm. (h) Detection of intracellular ROS via DCFH‐DA (10 µM) in MCF‐7 cells. MCF‐7 cells incubated with TPAPV‐NIM‐M and DCFH‐DA + light irradiation. (i) MCF‐7 cells incubated with Hoechst 33342 and DCFH‐DA + light irradiation. Light intensity (~27 mW/cm^2^). Scale bar: 20 µm.

TPAPV‐NIM‐M is an amphiphilic molecule decorated with a quaternary ammonium unit; it exhibits a zeta potential of +23.5 mV (Figure 3a) and forms uniform‐sized nanoaggregates (∼100 nm) in PBS with good stability, according to dynamic light scattering analysis (Figure 3b,c). It was expected that it would have strong PM interactions, as the hydrophilic moiety of TPAPV‐NIM‐M (quaternary ammonium unit) can form strong electrostatic interactions with the negatively charged PM, and the hydrophobic moiety can induce strong hydrophobic interactions with the hydrophobic lipid tails [42]. To substantiate our postulate, MCF‐7 breast cancer cells were incubated with 5 µM of TPAPV‐NIM‐M for 30 min and observed under a confocal laser scanning microscope. Interestingly, TPAPV‐NIM‐M stained the PM with strong red fluorescence, suggesting a high affinity of TPAPV‐NIM‐M for the PM. To further evaluate the PM specificity of TPAPV‐NIM‐M, two other cancer cell lines (MDA‐MB‐231 and Saos‐2) were incubated with TPAPV‐NIM‐M (5 µM) for 30 min and observed under a confocal laser scanning microscope. TPAPV‐NIM‐M localized at the PM of MDA‐MB‐231 and Saos‐2 cells with strong red fluorescence from the PM, confirming the high affinity of the synthesized TPAPV‐NIM‐M for the PM (Figure 3d). The PM specificity of TPAPV‐NIM‐M was further assessed co‐staining, where MCF‐7 cells were co‐incubated with TPAPV‐NIM‐M and the commercial PM dyes, WGA‐conjugates (5 µM) or DiO (10 µM), and observed under a confocal laser scanning microscope. The red fluorescence signals from TPAPV‐NIM‐M overlapped well with the green signals from WGA‐conjugates and DiO (Figure 3e), and the Pearson's correlation coefficients (PCCs) were calculated to be 0.804 for WGA‐conjugates and 0.793 for DiO (Figure 3e), confirming the high specificity of TPAPV‐NIM‐M for the PM. For the PM‐specific PDT, a PS must remain at the PM for a longer period. To evaluate the retention ability of TPAPV‐NIM‐M at the PM, the time‐dependent retention/internalization of TPAPV‐NIM‐M was studied in MCF‐7 cells. MCF‐7 cells were incubated with 5 µM TPAPV‐NIM‐M for 10–300 min and then observed using a confocal laser scanning microscope. TPAPV‐NIM‐M stained the PM within 10 min and was retained at the PM for 300 min with a slight internalization into the cytoplasm, confirming the high affinity of TPAPV‐NIM‐M for the PM (Figure 3f). The retention time of TPAPV‐NIM‐M at the PM was further evaluated over 24 h, and it anchored to the PM, with intense red fluorescence emanating from the PM even after 24 h of incubation (Figure 3g), further supporting the high affinity of TPAPV‐NIM‐M for the PM. Next, we evaluated the photostability of TPAPV‐NIM‐M, another critical parameter in bioimaging for long‐term visualization and tracking of cellular processes. The photostability of TPAPV‐NIM‐M was assessed under continuous laser scanning at 10% laser power for 5 min using the MCF‐7 cells (Figure S7), where the fluorescence signal loss was negligible, indicating the high photostability of TPAPV‐NIM‐M.

### 2.4 In Vitro Photoinduced Therapy of Cancer Cells

Inspired by the excellent PM interactions, long retention time at the PM, and high ROS generation in aqueous medium, we further investigated the application of TPAPV‐NIM‐M in photodynamic breast cancer therapy. For PDT of cancer cells, a PS should generate ROS inside cells; therefore, we first evaluated the intracellular ROS‐generating ability of TPAPV‐NIM‐M using 2′,7′‐dichlorofluorescein diacetate (DCFH‐DA), a standard commercial ROS sensor. Upon cellular uptake, DCFH‐DA, a nonfluorescent molecule, is deacetylated by intracellular esterases to the nonfluorescent compound DCFH, which then oxidizes to intense green‐emitting DCF in the presence of ROS [68, 69]. For intracellular ROS detection, MCF‐7 cells were incubated with 5 µM TPAPV‐NIM‐M for 2 h in a glass‐bottom confocal dish, then washed with PBS, treated with DCFH‐DA (10 µM), and incubated for 30 min. After 30 min, the cells were washed twice with PBS, irradiated with white light (∼27 mW/cm^2^) for 10 min, and immediately observed under a confocal microscope. Intense green fluorescence signals were observed in MCF‐7 cells (Figure 3h), which were attributed to the oxidation of DCFH to DCF by ROS generated by TPAPV‐NIM‐M. Cells incubated only with DCFH‐DA did not exhibit any green fluorescence signal in MCF‐7 cells upon light irradiation (Figure 3i), indicating that TPAPV‐NIM‐M has the ability to generate intracellular ROS, which is vital for the PDT of cancer cells.

**FIGURE 4:**
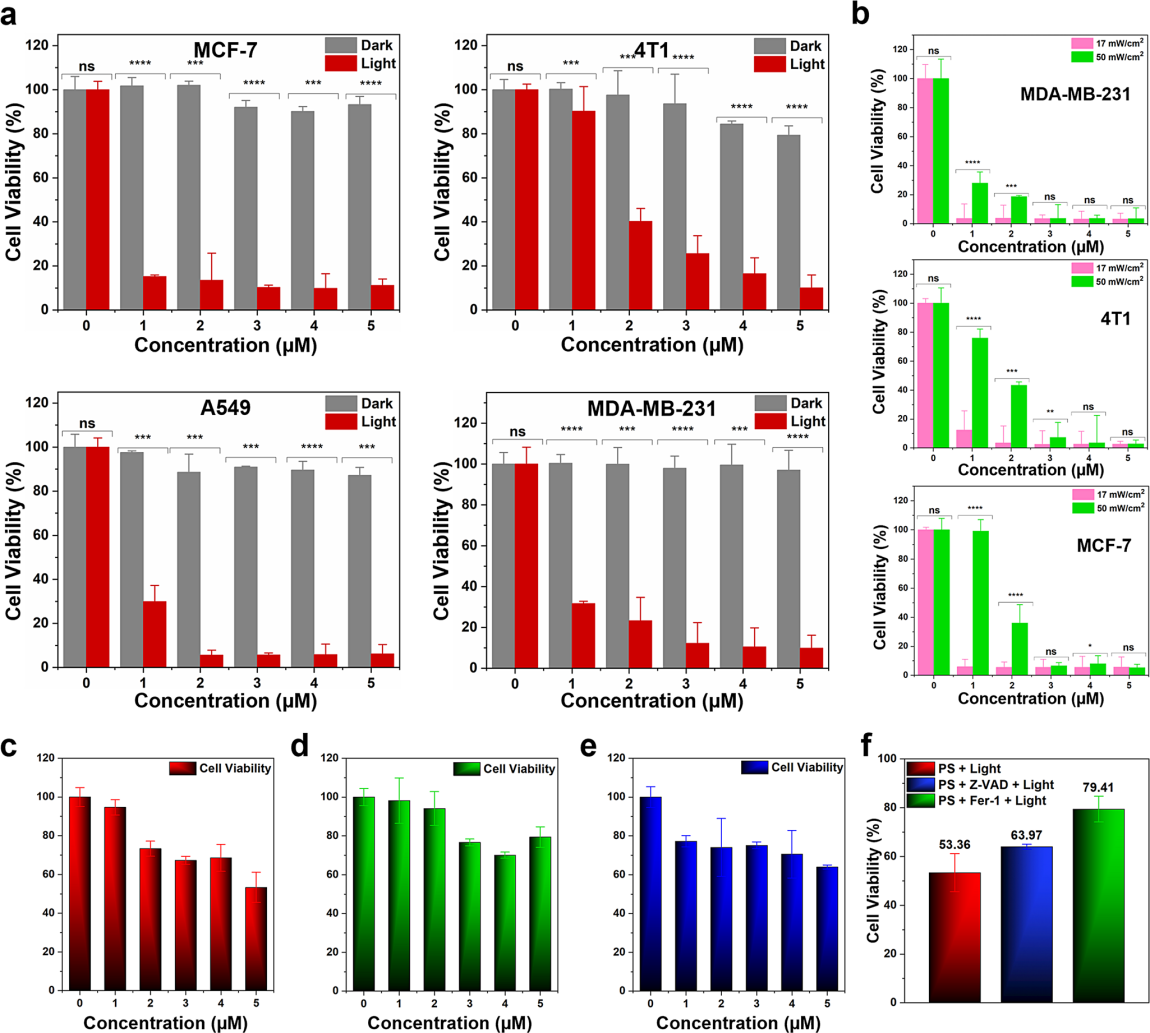
(a) Cell viability of MCF‐7, A549, 4T1, and MDA‐MB‐231 cells incubated with different concentrations of TPAPV‐NIM‐M (0–5 µM) in the dark and under white‐light irradiation for 20 min (∼27 mW/cm^2^). Data are presented as the mean ± SD (*n* = 6). (b) Cell viability of MDA‐MB‐231, 4T1, and MCF‐7 cells incubated with TPAPV‐NIM‐M (0–5 µM) under white‐light irradiation at various light intensities (17 and 50 mW/cm^2^). (c) Cell viability of MDA‐MB‐231 cells incubated with TPAPV‐NIM‐M (0–5 µM) under white‐light irradiation (27 mW/cm^2^) for 5 min. (d) Cell viability of MDA‐MB‐231 cells incubated with TPAPV‐NIM‐M (0–5 µM) under white‐light irradiation (27 mW/cm^2^) for 5 min in the presence of Fer‐1 (20 µM). (e) Cell viability of MDA‐MB‐231 cells incubated with TPAPV‐NIM‐M (0–5 µM) under white‐light irradiation (27 mW/cm^2^) for 5 min in the presence of Z‐VAD (40 µM). (f) Comparative cell viability of MDA‐MB‐231 cells treated with TPAPV‐NIM‐M (5 µM) under white‐light irradiation (27 mW/cm^2^) for 5 min in the absence or presence of Z‐VAD (40 µM) and Fer‐1 (20 µM). PS denotes TPAPV‐NIM‐M. Statistical significance was determined by the Student's *t*‐test as follows: *p < 0.05, **p < 0.01, ***p < 0.001, ****p < 0.0001, and ns (not significant).

Encouraged by the excellent intracellular ROS‐generating ability of TPAPV‐NIM‐M, we further evaluated its efficacy for in vitro photodynamic ablation of cancer cells. A549, MDA‐MB‐231, and 4T1 cells were separately incubated with different concentrations (0–5 µM) of TPAPV‐NIM‐M for 2 h, then irradiated with white light (∼27 mW/cm^2^) for 20 min, followed by a further 12 h incubation; cell viability was then assessed with the standard MTT assay. Compared with the TPAPV‐NIM‐M‐untreated cells (control group), cell viability decreased as PS concentration increased (Figure 4a). The cell viability of MCF‐7 cells was 15% at 1 µM and decreased to 10% at 5 µM, indicating a strong cytotoxic effect under white‐light irradiation. Similarly, the cell viabilities of A549, 4T1, and MDA‐MB‐231 cells were 30%, 90%, and 32% at 1 µM, respectively, and decreased to 6%, 10%, and 10% at 5 µM, respectively, further supporting a potent cytotoxic effect of TPAPV‐NIM‐M under white‐light irradiation. Similarly, the dark toxicity of TPAPV‐NIM‐M was also investigated in the absence of light, and the results presented in Figure 4a indicated that TPAPV‐NIM‐M exhibited negligible dark toxicity at the working concentration (5 µM).

The effect of light intensity on cell viability was also investigated in MDA‐MB‐231, 4T1, and MCF‐7 cells. To evaluate this effect, MDA‐MB‐231, 4T1, and MCF‐7 cells were separately incubated with various TPAPV‐NIM‐M concentrations (0–5 µM) for 2 h. The cells were then irradiated under white light at intensities of 17 and 50 mW/cm^2,^ and dose‐dependent phototoxicity was observed (Figure 4b). These results suggest that TPAPV‐NIM‐M has high potential to induce phototoxicity in cancer cells under white‐light irradiation and is a suitable candidate for PM‐targeted antitumor PDT.

To verify the pathways of cell death, inhibitors of apoptosis and ferroptosis were employed. Ferrostatin‐1 (Fer‐1) is a potent ferroptosis inhibitor that inhibits lipid peroxidation and ROS generated by a PS under light irradiation, while Z‐VAD‐FMK (Z‐VAD) is a pan‐caspase inhibitor that inhibits apoptosis [70, 71, 72]. To evaluate cell death pathways, Fer‐1 and Z‐VAD‐pretreated MDA‐MB‐231 cells were incubated with varying concentrations (0–5 µM) of TPAPV‐NIM‐M for 2 h, irradiated with white light (27 mW/cm^2^) for 5 min, incubated for a further 24 h, and cell viability was then measured using an MTT assay. Cell viability in Fer‐1‐pretreated cells was higher than in irradiated cells without Fer‐1 treatment (Figure 4c), indicating that Fer‐1 significantly protected cells from photodynamic damage (Figure 4d), which strongly supports the involvement of ferroptosis in the photodynamic ablation of cancer cells. Moreover, cell viability in Z‐VAD‐pretreated cells was also higher than that in the light groups without Z‐VAD treatment (Figure 4e,f), indicating the involvement of apoptosis in cell death. The high phototoxicity of TPAPV‐NIM‐M in cancer cells may result from the combined contributions of apoptosis and ferroptosis, which is an effective strategy for improving PDT performance [73].

### 2.5 Live/Dead Cell Staining

**FIGURE 5:**
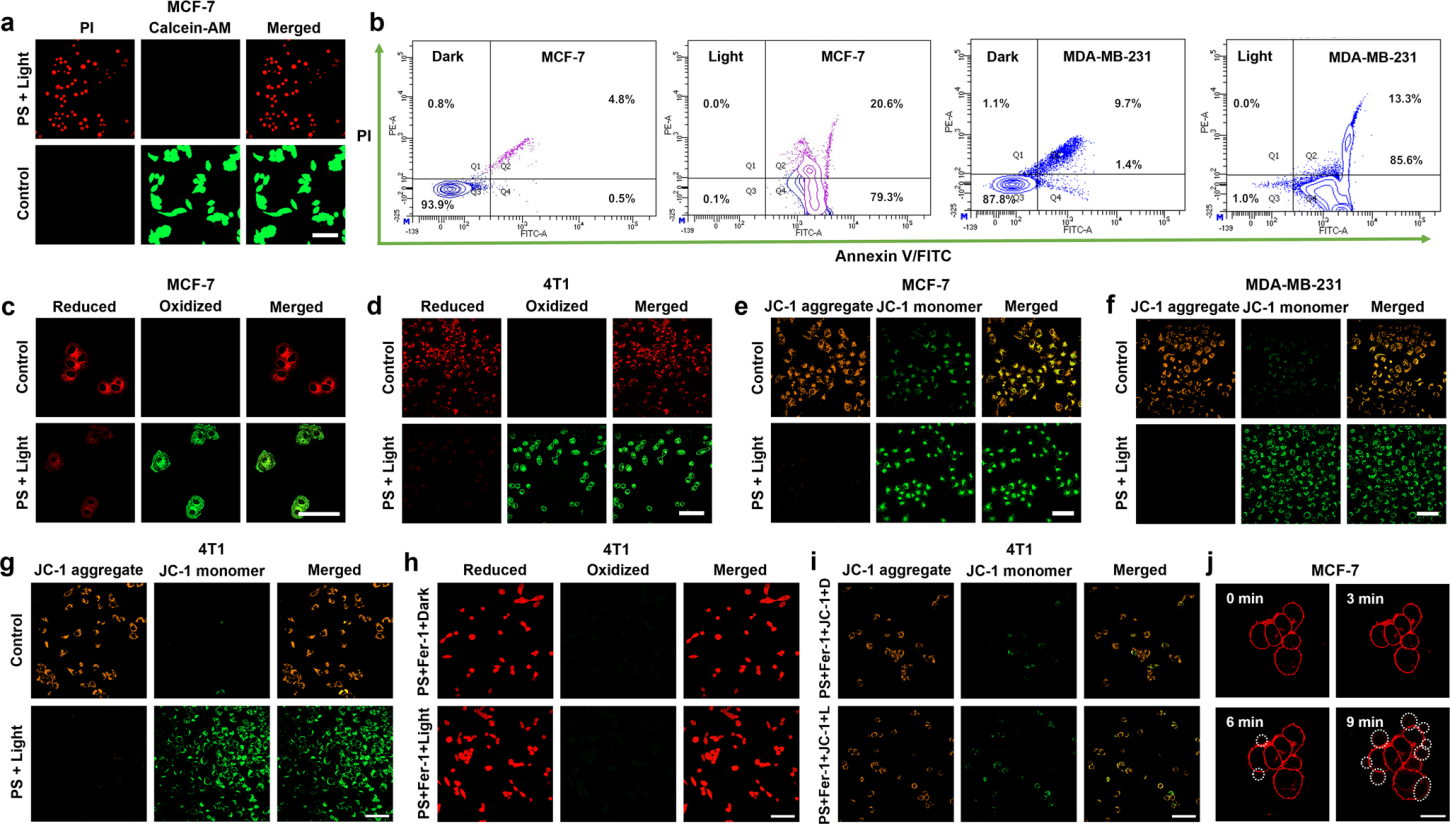
(a) Live/dead cell assay in MCF‐7 cells treated with TPAPV‐NIM‐M (5 µM) in the presence of white‐light irradiation (∼27 mW/cm^2^), and the control group was treated with calcein‐AM/PI. Live cells are labeled by calcein‐AM with green fluorescence, and dead cells are labeled by PI with red fluorescence. Calcein‐AM was excited with a 488 nm laser, and the emission was collected between 450 and 555 nm. PI was excited with a 561 nm laser, and the emission was collected between 450 and 700 nm. Scale bar: 20 µm. (b) Apoptosis tracking in MCF‐7 cells and MDA‐MB‐231 cells: dark (MCF‐7 or MDA‐MB‐231 cells only treated with Annexin V‐FITC/PI in the dark) and light (MCF‐7 or MDA‐MB‐231 cells treated with TPAPV‐NIM‐M and Annexin V‐FITC/PI under light) (∼27 mW/cm^2^). (c,d) Lipid peroxide (LPO) detection in MCF‐7 and 4T1 cells via C11‐BODIPY‐581/591. Scale bars: 10 and 20 µm in (c) and (d), respectively. The reduced form was excited with a 561 nm laser, and the emission was collected between 575 and 620 nm. The oxidized form was excited via a 488 nm laser, and the emission was collected between 450 and 560 nm. (e–g) Detection of changes in the MMP in MCF‐7, MDA‐MB‐231, and 4T1 cells under light and dark conditions using JC‐1. Scale bars: 20 µm. JC‐1 aggregate: excited with a 561 nm laser, and the emission was collected between 450 and 700 nm. JC‐1 monomer: excited with a 488 nm laser, and the emission was collected between 490 and 560 nm. (h) Detection of LPO in 4T1 cells via C11‐BODIPY‐581/591 in the presence of Fer‐1 (5 µM). Scale bar: 20 µm. (i) Detection of changes in the MMP in 4T1 cells under light and dark conditions by using JC‐1 in the presence of Fer‐1 (5 µM). Scale bar: 20 µm. D stands for dark. (j) Real‐time tracking of apoptosis induced by PS in MCF‐7 cells under laser irradiation for 9 min. Scale bar: 10 µm. PS denotes TPAPV‐NIM‐M.

To evaluate the effectiveness of PDT induced by TPAPV‐NIM‐M, a live/dead cell‐staining assay was performed using a calcein acetoxymethyl ester (calcein‐AM)/propidium iodide (PI) kit. Calcein‐AM is nonfluorescent and, upon cellular uptake, is hydrolyzed by the intracellular esterases to green fluorescent calcein, which is commonly used to differentiate live cells from dead cells, while PI enters the nuclei of dead cells and emits red fluorescence [74]. MCF‐7 cells were incubated with TPAPV‐NIM‐M (5 µM) for 2 h in a glass‐bottom confocal dish; thereafter, the light group was irradiated with white light for 10 min, treated with a calcein‐AM/PI kit for 30 min, and observed under a confocal microscope. Intense green fluorescence signals were observed in the control group (dark group), indicating no dark toxicity from TPAPV‐NIM‐M, whereas in the light‐treated group, bright red fluorescence signals from PI were observed, indicating effective photodynamic ablation of cancer cells under light irradiation (Figure 5a). To gain further insight into the PDT of cancer cells under light irradiation (∼27 mW/cm^2^), the Annexin V‐FITC/PI kit was used to quantitatively assess TPAPV‐NIM‐M‐induced apoptosis via flow cytometry. To perform flow cytometry analysis, 1 × 10^6^ MCF‐7 and MDA‐MB‐231 cells were cultured separately in a glass‐bottom confocal dish and treated with TPAPV‐NIM‐M for 2 h; thereafter, the cells were stained with Annexin V‐FITC/PI according to the manufacturer's protocol and analyzed by flow cytometry, and the results showed that TPAPV‐NIM‐M induced apoptosis in MCF‐7 and MDA‐MB‐231 cells, further confirming its effectiveness for PDT (Figure 5b).

Inspired by long‐term PM retention, high ROS (type I) generation, and excellent light‐induced toxicity in cancer cells, we speculated that TPAPV‐NIM‐M may also induce ferroptosis, as PMs are rich in polyunsaturated fatty acids (PUFAs), which are necessary for ferroptosis [75]. To validate our hypothesis, a commercial lipid peroxide (LPO) sensor (C11‐BODIPY‐581/591) was used to detect cellular LPO, as its accumulation is strong evidence of ferroptosis, a programmed cell death induced by lipid peroxidation [76]. In the reduced state, C11‐BODIPY‐581/591 emits intense red fluorescence, while in the oxidized state, it emits green fluorescence. To evaluate whether TPAPV‐NIM‐M can induce ferroptosis in cancer cells, MCF‐7 and 4T1 cells were incubated with TPAPV‐NIM‐M (5 µM) for 2 h, followed by treatment with C11‐BODIPY‐581/591 (10 µM) for 30 min. The cells were washed with PBS, irradiated with white light (∼27 mW/cm^2^) for 10 min, and then observed under a confocal microscope. Green fluorescence signals were observed for the light‐treated groups, while red fluorescence signals were observed for the control groups, indicating that under white‐light irradiation, TPAPV‐NIM‐M can cause lipid peroxidation to generate LPO and trigger ferroptosis, which further supports the effectiveness of TPAPV‐NIM‐M in killing cancer cells via hybrid pathways (Figure 5c,d). To further verify ferroptosis, a mitochondrial membrane potential (MMP) assay was performed using JC‐1, a standard dye used to monitor changes in MMP. In healthy mitochondria, JC‐1 aggregates on the mitochondrial surface, producing red fluorescence, whereas in damaged/depolarized mitochondria, it fails to aggregate due to loss of MMP and instead exists as a monomer, producing green fluorescence [77]. To perform the MMP assay, MCF‐7, MDA‐MB‐231, and 4T1 cells were separately incubated with TPAPV‐NIM‐M (5 µM) in a glass‐bottom confocal dish for 2 h, washed with PBS, and irradiated with white light (∼27 mW/cm^2^) for 5 min. The light‐treated cells were treated with JC‐1 (3 µM) for 30 min, washed with PBS, and quickly observed under a confocal laser scanning microscope. Bright red fluorescence signals were observed in the control group, indicating healthy mitochondria, and in the light group, the light irradiation caused mitochondrial damage/depolarization due to ROS and LPO generation, resulting in the green fluorescence signals of JC‐1 monomer, which is a strong sign of ferroptosis (Figure 5e–g) [78, 79].

To further validate ferroptosis‐mediated cell death, Fer‐1‐pretreated 4T1 cells were incubated with TPAPV‐NIM‐M and C11‐BODIPY‐581/591. The cells were irradiated with white light (∼27 mW/cm^2^) for 5 min and observed under a confocal microscope. Fer‐1 effectively reduced the lipid ROS level, as evidenced by the suppression of green fluorescence in the light‐treated group (Figure 5h). To further validate the reduction in lipid ROS levels, JC‐1 staining assay was performed on Fer‐1‐pretreated 4T1 cells. In the presence of TPAPV‐NIM‐M and light irradiation, no significant change in MMP was observed, as evidenced by the intense red fluorescence of mitochondria (Figure 5i), indicating healthy mitochondria, further validating the effective reduction in lipid ROS, thereby confirming ferroptosis‐mediated cell death. Fer‐1‐pretreated 4T1 cells incubated with TPAPV‐NIM‐M and C11‐BODIPY‐581/591, or with JC‐1 without light treatment, served as the control groups. Taken together, these results indicate that TPAPV‐NIM‐M, with its high ROS‐ and LPO‐generating capacity, can induce both apoptosis and ferroptosis in cancer cells under low‐intensity white‐light irradiation and is a suitable candidate for eliminating tumors through multiple cell‐death pathways.

### 2.6 Tracking of Apoptosis in Real‐Time

Given its high ROS‐generating ability, excellent PM staining, long PM‐retention time, and high photostability, TPAPV‐NIM‐M was used to monitor apoptosis in MCF‐7 cells in real time. MCF‐7 cells were incubated with TPAPV‐NIM‐M for 1 h and then subjected to sequential laser irradiation while confocal images were acquired. The representative images in Figure 5j summarize the morphological evolution at cumulative irradiation times of 0, 3, 6, and 9 min, whereas Figures S8 and S9 provide detailed time‐lapse snapshots at 20 s intervals for the 3–6 and 6–9 min irradiation periods, respectively. During the initial 3 min of laser irradiation, TPAPV‐NIM‐M remained localized at the PM and no obvious apoptotic morphology was observed. When the irradiation time was extended to 6 min, membrane blebs began to appear on the PM, indicating the onset of apoptosis. Further irradiation for up to 9 min resulted in the formation of more numerous and larger PM blebs, confirming the progressive development of apoptosis. These time‐dependent morphological changes demonstrate that TPAPV‐NIM‐M not only induces apoptosis in cancer cells but also self‐reports the process through PM‐localized fluorescence, enabling real‐time tracking of apoptosis. Notably, TPAPV‐NIM‐M remained anchored to the PM throughout irradiation without diffusing into the cytoplasm, and negligible fluorescence loss was observed even at 40% laser intensity, further confirming its strong PM affinity and high photostability.

### 2.7 Bacterial Imaging

**FIGURE 6:**
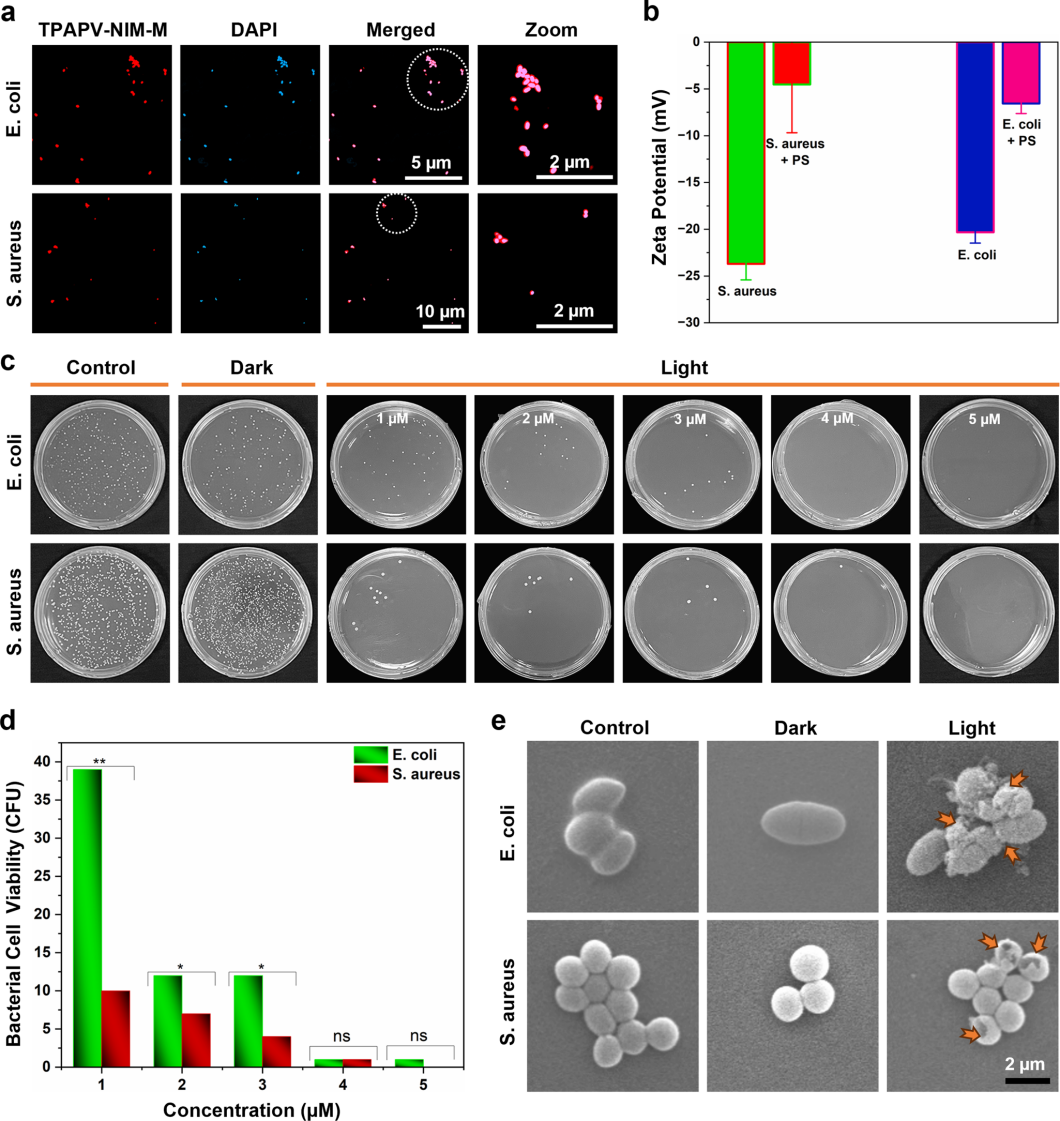
(a) Confocal fluorescence images of *E*. *coli* and *S*. *aureus* treated with TPAPV‐NIM‐M (red fluorescence signals), DAPI (blue fluorescence signals), and merged images. Concentration of TPAPV‐NIM‐M: 5 µM and DAPI: 20 µM. DAPI was excited with a 405 nm laser, and the emission was collected between 400 and 560 nm. (b) Zeta potential values of *S*. *aureus* and *E*. *coli* with and without treatment with TPAPV‐NIM‐M (5 µM). (c) Survival rates of *E*. *coli* and *S*. *aureus* under different treatments: control (only *E*. *coli* or *S*. *aureus* + light), dark (*E*. *coli* or *S*. *aureus* treated with TPAPV‐NIM‐M under dark conditions), and light (*E*. *coli* or *S*. *aureus* treated with TPAPV‐NIM‐M (0–5 µM) + light). d) Graphical representation of the bacterial viability of *E*. *coli* and *S*. *aureus* under different treatments with TPAPV‐NIM‐M. The light intensity was ∼40 mW/cm^2^. Data are presented as the mean ± SD (*n* = 5). Statistical significance was determined by the Student's *t*‐test as follows: *p < 0.05, **p < 0.01, ***p < 0.001, ****p < 0.0001, and ns (not significant). (e) SEM images of *E*. *coli* and *S*. *aureus* after different treatments: control (only *E*. *coli* and *S*. *aureus* + light), dark (*E*. *coli* and *S*. *aureus* treated with TPAPV‐NIM‐M in the dark), and light (*E*. *coli* and *S*. *aureus* treated with TPAPV‐NIM‐M and irradiated with white light). The light intensity was ∼40 mW/cm^2^.

Amphiphilic organic molecules are ideal candidates for strong interactions with negatively charged bacterial surfaces. TPAPV‐NIM‐M is an amphiphilic molecule and was therefore expected to interact strongly with bacteria. To verify this, we used two bacterial strains: *Staphylococcus aureus* (*S. aureus*), a G^+^ bacterium, and *Escherichia coli* (*E. coli*), a G^−^ bacterium. To evaluate the ability of TPAPV‐NIM‐M to interact with bacteria, *S*. *aureus* and *E*. *coli* were treated with TPAPV‐NIM‐M (5 µM) for 20 min at 37°C under shaking conditions, then washed with PBS, and subsequently treated with DAPI (20 µM) for 10 min. After washing with PBS, bacteria were observed under a confocal laser scanning microscope. Interestingly, bright red fluorescence signals were observed in both *S*. *aureus* and *E*. *coli*, along with blue DAPI signals from the bacterial DNA (Figure 6a). The red fluorescence signals from TPAPV‐NIM‐M overlapped well with the blue signals from DAPI, indicating that TPAPV‐NIM‐M not only stains the bacterial membrane but also diffuses into the protoplasm, suggesting strong binding to both *S*. *aureus* and *E*. *coli*. The affinity of TPAPV‐NIM‐M for bacterial surfaces was further investigated using a Zetasizer. After *S*. *aureus* and *E. coli* were incubated with TPAPV‐NIM‐M for 20 min, their zeta potentials were measured using a Zetasizer. Incubation with TPAPV‐NIM‐M shifted the zeta potentials of *S. aureus* and *E. coli* from −23.7 to −4.52 mV and from −21.0 to −6.18 mV, respectively (Figure 6b), indicating charge neutralization through strong interactions with the negatively charged bacterial surfaces.

### 2.8 In Vitro Photodynamic Bacterial Ablation

Given its excellent bacterial staining capability, strong interactions with bacterial surfaces, and high ROS‐generating ability, we further assessed TPAPV‐NIM‐M for in vitro antibacterial PDT against *E*. *coli* and *S*. *aureus* using the plate counting method. To assess the concentration‐dependent phototoxicity of TPAPV‐NIM‐M, bacterial suspensions with an OD_600_ = 0.1 were incubated with different concentrations (1–5 µM) of TPAPV‐NIM‐M in PBS for 20 min, then washed with PBS and irradiated with white light (∼40 mW/cm^2^) for 20 min. For comparison, bacterial suspensions (OD_600_ = 0.1) without treatment with TPAPV‐NIM‐M were also irradiated under white light (∼40 mW/cm^2^) for 20 min, and to assess the dark toxicity of TPAPV‐NIM‐M, *E*. *coli* and *S*. *aureus* suspensions in PBS (OD_600_ = 0.1) were also incubated with TPAPV‐NIM‐M (5 µM) for 20 min and used without light treatment. To evaluate dark and light toxicities, light‐treated and untreated groups were serially diluted to 1 × 10^4^ CFU, and 70 µL aliquots of the suspension were evenly spread on LB agar plates and incubated at 37°C for 24 h. TPAPV‐NIM‐M induced concentration‐dependent phototoxicity with nearly 100% bacterial elimination ability at 5 µM, indicating strong antibacterial efficacy against both G^−^ and G^+^ bacterial strains. In addition, TPAPV‐NIM‐M had negligible dark toxicity against both *S*. *aureus* and *E*. *coli* (Figure 6c,d). The photoinduced inactivation of bacteria was further studied using scanning electron microscopy (SEM). After PDT with TPAPV‐NIM‐M under white‐light irradiation (∼40 mW/cm^2^) for 20 min, the cell walls of both *S*. *aureus* and *E*. *coli* were severely damaged, as evidenced by the rough and ruptured surfaces compared with the smooth surfaces of the control groups (Figure 6e), which further supports the strong phototoxicity of TPAPV‐NIM‐M against G^+^ and G^−^ bacteria under white‐light irradiation (∼40 mW/cm^2^).

### 2.9 In Vivo Wound‐Infection Model

**FIGURE 7:**
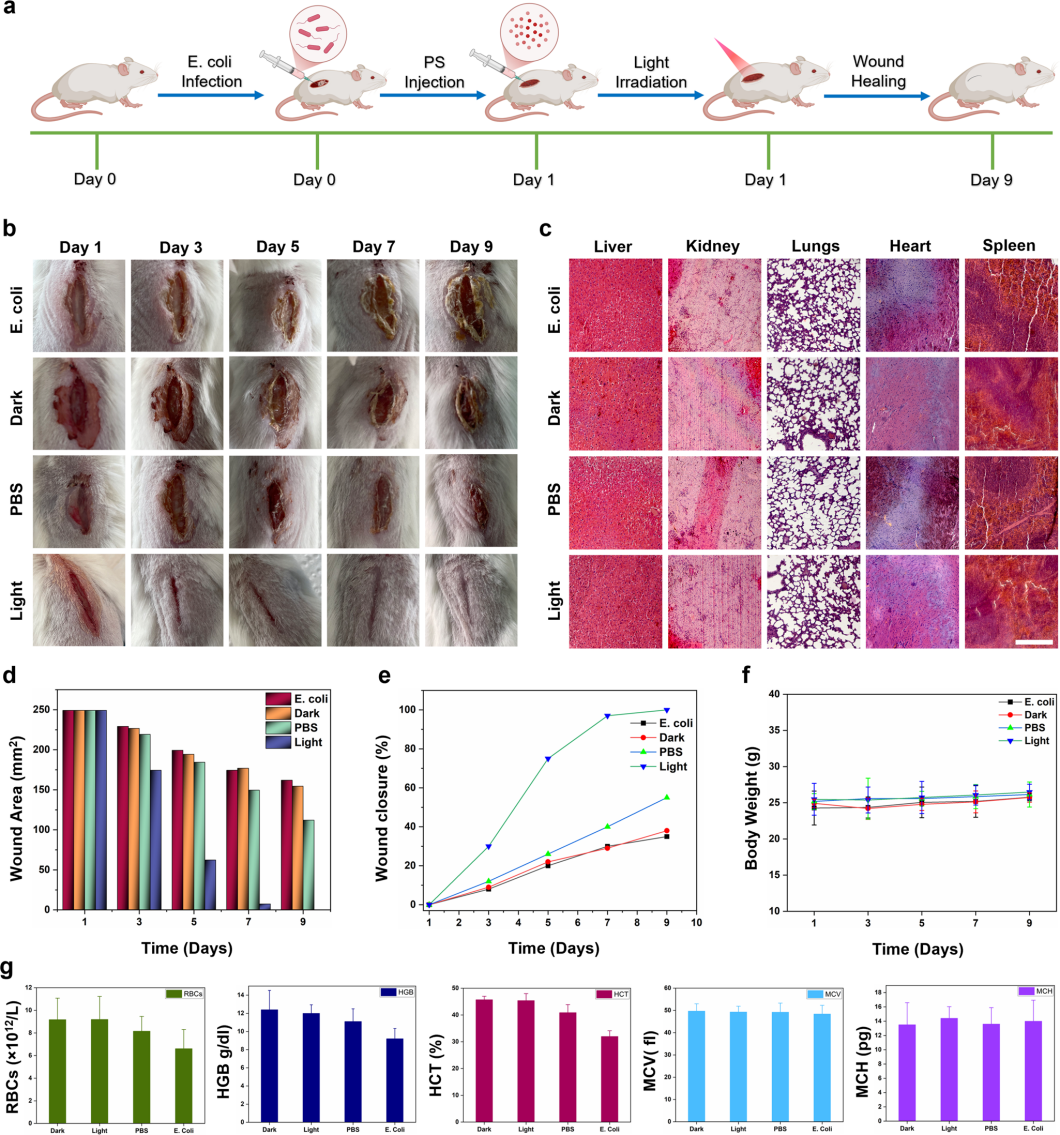
(a) Experimental design and schedule for the anti‐infection wound‐healing model. (b) Photographs of *E*. *coli*‐infected BALB/c mouse wounds with different treatments: *E*. *coli* (only *E*. *coli*), dark (*E*. *coli* treated with TPAPV‐NIM‐M (20 µM) in the dark), PBS (*E*. *coli* treated with PBS and white‐light irradiation at ∼85 mW/cm^2^), and light (*E*. *coli* treated with TPAPV‐NIM‐M (20 µM) under white‐light irradiation at ∼85 mW/cm^2^). (c) H&E staining images of vital organs (liver, kidney, lungs, heart, and spleen) after nine days of various treatments in *E*. *coli*‐infected BALB/c mice. Scale bar: 50 µm. (d) Graphical representation of the wound areas (mm^2^) of the mice under different treatments over 9 days of the experimental period. (e) Graphical representation of the wound closure (%) of the mice under different treatments over the 9 day experimental period. (f) Body‐weight profiles of mice in the different treatment groups over the 9 day experimental period. (g) Hematological parameters of the blood samples collected from mice of the different treatment groups after nine days of treatment.

Inspired by the excellent in vitro antibacterial activity of TPAPV‐NIM‐M, we further investigated its in vivo antibacterial PDT efficacy using an *E*. *coli*‐infected wound model in BALB/c mice. To evaluate the wound‐healing ability, a linear dorsal skin incision (∼20 mm in length) was created on the back of each mouse (Figure 7a). The mice were then randomly divided into four groups (*n* = 3): (1) *E*. *coli* group (only *E*. *coli*), (2) dark group (*E*. *coli* + TPAPV‐NIM‐M), (3) PBS group (*E*. *coli* + PBS + light), and (4) light group (*E*. *coli* + TPAPV‐NIM‐M + light). At 24 h post‐infection with *E*. *coli*, groups (2) and (4) were treated with 20 µM TPAPV‐NIM‐M and kept at room temperature for 2 h, while group (3) received only PBS treatment. After 2 h, groups (3) and (4) were irradiated with white light (∼85 mW/cm^2^) for 25 min, and the wound‐healing progress was monitored photographically. Scabs appeared on the wounds of the *E*. *coli*, PBS, and dark groups by day 3, and continued to thicken by day 9. Similarly, ulcers and exudates also appeared around the wounds in the control groups, indicating severe wound infection. In contrast, no scabs, ulcers, or exudates formed in the light group, and the wounds began to heal by day 3 and were completely healed by day 7 (Figure 7b), indicating superior photodynamic wound‐healing efficiency (Figure 7d,e). In addition, the body weights of the mice were monitored throughout the experiment, and no abnormal changes were observed (Figure 7f), indicating good biosafety for in vivo applications.

Biocompatibility is a critical parameter in determining the biosafety of a PS for biomedical applications. To evaluate whether TPAPV‐NIM‐M induced adverse effects in the treated mice, hematoxylin and eosin (H&E) staining was performed on vital organs (liver, spleen, heart, lungs, and kidneys) collected from the different treatment groups. On day 9 of the experiment, blood was collected from the treated mice in each group. Subsequently, the mice were euthanized, and their vital organs were harvested for H&E analysis. H&E analysis revealed no noticeable inflammatory lesions or tissue damage in the major organs (Figure 7c). Similarly, the basic blood parameters, such as RBCs, HGB, HCT, MCV, and MCH, were within their respective reference ranges, indicating the high biocompatibility of TPAPV‐NIM‐M (Figure 7g). In addition, the hemolytic effect of TPAPV‐NIM‐M on RBCs collected from a healthy mouse was investigated, with deionized water as a positive control (100% hemolysis) and PBS as a negative control (no hemolysis). TPAPV‐NIM‐M did not induce hemolysis, even at a high concentration (100 µM) (Figure S10). Based on these biosafety results, we conclude that TPAPV‐NIM‐M has a favorable biosafety profile and can effectively treat bacterial infections under white‐light irradiation.

### 2.10 In Vivo Antitumor PDT

**FIGURE 8:**
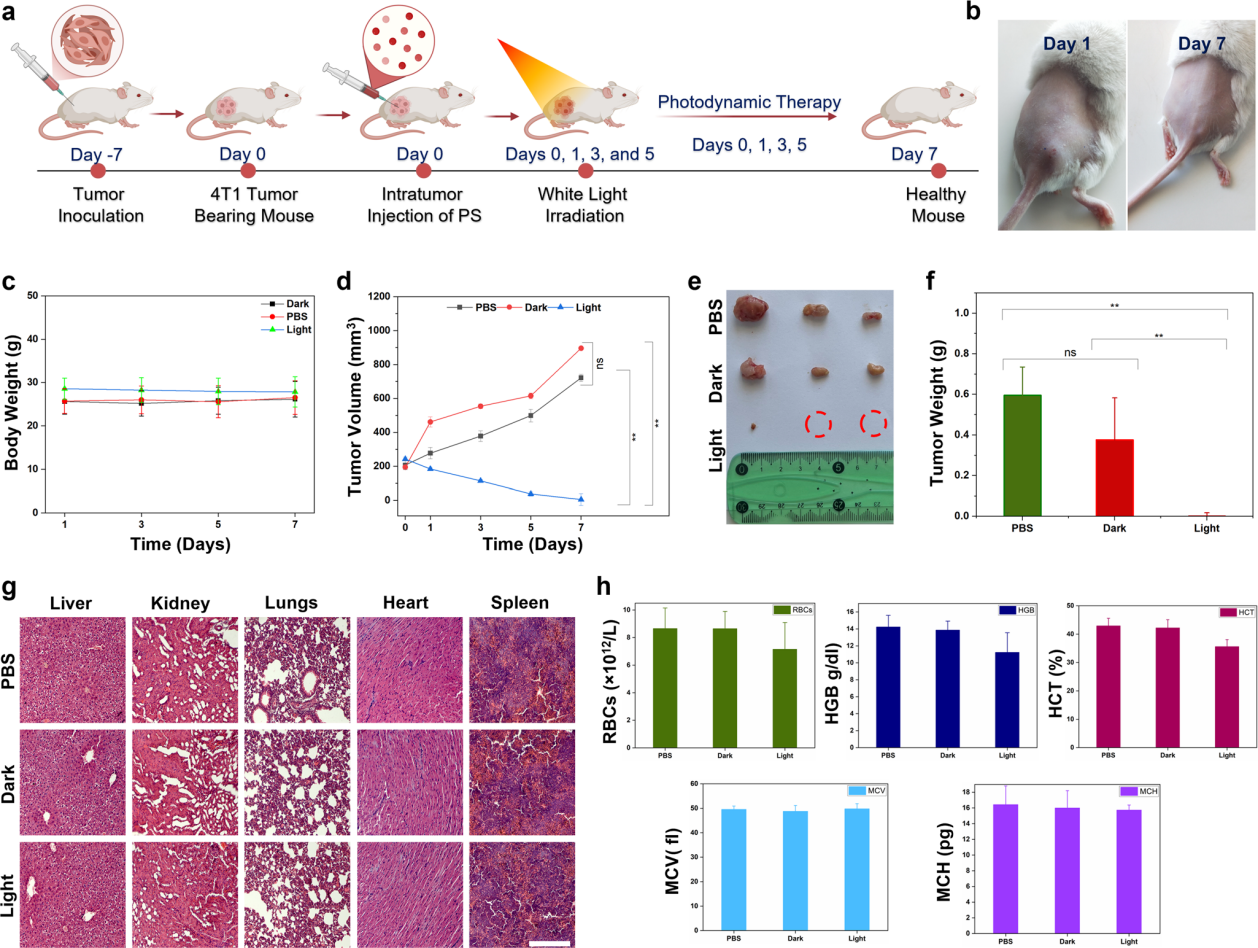
(a) Experimental design and schedule for the antitumor PDT of 4T1 tumor‐bearing BALB/c mice. (b) Photographs of the 4T1 tumor‐bearing mice before and after the PDT. (c) Body weight of the mice under different antitumor PDT treatments over 7 days. (d) Changes in the tumor volume of different treatment groups during the antitumor PDT experiment. (e) Photographs of the tumors excised from mice in all groups on day 7 of the treatment. (f) Average weight of the tumors excised from the different treatment groups at the end of the PDT experiment. (g) H&E staining images of vital organs (liver, kidney, lungs, heart, and spleen) after seven days of various treatments of the 4T1 tumor‐bearing BALB/c mice. Scale bar: 50 µm. (h) Hematological parameters of the blood samples collected from mice of the different treatment groups after seven days of treatment. Data are presented as the mean ± SD (*n* = 3). Statistical significance was determined by the Student's *t*‐test as follows: *p < 0.05, **p < 0.01, ***p < 0.001, ****p < 0.0001, and ns (not significant).

Encouraged by the high ROS‐generating capacity and excellent in vitro photodynamic ablation of cancer cells, the antitumor efficacy of TPAPV‐NIM‐M was further investigated in 4T1 tumor‐bearing female BALB/c mice. The experimental design and treatment schedule are shown in Figure 8a. To evaluate the in vivo antitumor phototoxicity of TPAPV‐NIM‐M, 4T1 tumor‐bearing mice were randomly divided into three groups: the PBS group (treated with PBS under light irradiation), the dark group (treated with TPAPV‐NIM‐M), and the light group (treated with TPAPV‐NIM‐M under light irradiation). The dark and light groups were treated with 100 µM TPAPV‐NIM‐M via intratumoral injection, while the PBS group received 100 µL of PBS. After 2 h, the light group was irradiated with white light (∼85 mW/cm^2^) for 25 min. This procedure was repeated on days 1, 3, and 5, and nearly complete tumor ablation was achieved by day 7 (Figure 8b). The body weights of the mice were monitored regularly, and no abnormal weight loss was observed during the experimental period (Figure 8c). Tumor sizes in the PBS and dark groups increased over time, whereas in the light‐treated group, tumor sizes decreased, indicating a strong tumoricidal effect of TPAPV‐NIM‐M under white‐light irradiation (Figure 8d). On day 7, blood was collected from the treated mice in each group. Thereafter, the mice were euthanized, and the tumors were excised, photographed (Figure 8e), and weighed (Figure 8f). The vital organs, including the liver, spleen, heart, lungs, and kidneys, were collected for H&E analysis, and no noticeable tissue damage was observed in any of the organs (Figure 8g). Based on hematological analysis of blood samples collected from mice in different experimental groups, the basic blood parameters, including RBCs, HGB, HCT, MCV, and MCH, were within their respective reference ranges, indicating the biocompatibility of TPAPV‐NIM‐M (Figure 8h). Taken together with the antibacterial findings, these results indicate that TPAPV‐NIM‐M, with a high biosafety profile, can treat bacterial infections and also effectively eliminate tumors, providing a novel platform for treating bacterial infections and eliminating tumors under white‐light irradiation.

## 3 Conclusion

In conclusion, three novel naphthalimide‐based AIE PSs, TPA‐NIM‐M, TPAV‐NIM‐M, and TPAPV‐NIM‐M, with D–A characteristics, were synthesized via Suzuki and Heck cross‐coupling reactions. Their chemical structures were characterized by nuclear magnetic resonance (NMR) spectroscopy and high‐resolution mass spectrometry. The synthesized PSs exhibited strong absorbance in the visible region, far‐red/NIR emission, and AIE in DMSO/toluene mixtures. Among these, TPAPV‐NIM‐M exhibited the largest Stokes shift (214 nm) and highest ROS generation, and was evaluated for anticancer and antibacterial PDT. It demonstrated strong cellular interactions, rapidly stained the PM of mammalian cells, anchored to the PM for extended periods (24 h) with minimal diffusion into the cell cytoplasm, and enabled real‐time tracking of apoptosis. In addition to inducing apoptosis, it also triggered ferroptosis in cancer cells under light irradiation. It also stained both G^−^ *E*. *coli* and G^+^ *S*. *aureus* because TPAPV‐NIM‐M is an amphiphilic molecule bearing a quaternary ammonium unit, which enhances its interactions with the negatively charged bacterial surface. According to theoretical calculations, TPAPV‐NIM‐M exhibited a narrow singlet‐triplet energy gap, facilitating intersystem crossing and thereby generating higher ROS under white‐light illumination. Due to strong cellular interactions and high ROS generation, TPAPV‐NIM‐M effectively eliminated both cancer and bacterial cells under white‐light irradiation, demonstrating high biocompatibility. It demonstrated excellent in vivo antibacterial PDT, as evidenced by rapid wound healing in BALB/c mice under white‐light irradiation. It also demonstrated a strong tumoricidal effect, with complete tumor elimination under white‐light irradiation in 4T1 tumor‐bearing BALB/c mice. Because of its excellent cellular interactions, high ROS generation, and efficient elimination of tumor cells and pathogenic bacteria under white‐light irradiation, TPAPV‐NIM‐M offers a novel strategy for designing next‐generation PSs with dual targeting of cancer and bacterial cells, enabling effective elimination of tumor tissues and pathogens.

## 4 Experimental Section

### 4.1 Synthetic Procedures

Synthetic procedures for TPA‐NIM‐M and TPAV‐NIM‐M are described in the Supporting Information. The NMR and MS spectra are shown in Figures S11–S25, and the synthetic routes of TPA‐NIM‐M, TPAV‐NIM‐M, and TPAPV‐NIM‐M are presented in Scheme S1.

#### 4.1.1 Synthesis of TPAPV‐NIM

TPAPV‐NIM was synthesized via a Heck cross‐coupling reaction. DAP‐NIM (0.150 g, 0.415 mmol), TPA‐PV (0.144 g, 0.415 mmol), palladium(II) acetate (0.01 g, 0.046 mmol), and tri(o‐tolyl)phosphine (0.025 g, 0.083 mmol) were added to a Schlenk flask, and the flask was degassed multiple times. Dry acetonitrile (15 mL) and triethylamine (0.81 mL) were added. The mixture was purged with nitrogen and refluxed at 90°C for 48 h. Thereafter, acetonitrile was removed under reduced pressure, and the resulting reaction mixture was taken up in dichloromethane (DCM), washed with deionized water and brine, dried over anhydrous sodium sulfate, filtered, and concentrated on a rotary evaporator. The resulting mixture was purified by column chromatography on silica gel using a 1:1 mixture of DCM and methanol, affording TPAPV‐NIM as a dark‐yellow solid (0.134 g, 51.43% yield). ^1^H NMR (DMSO‐*d6*, 400 MHz): *δ*/ppm = 1.76 (m, 2H), 2.13 (s, 6H), 2.29 (m, 2H), 4.04 (t, *J* = 8 Hz, 2H), 7.06 (m, 8H), 7.33 (m, 4H), 7.56 (d, *J* = 16 Hz, 1H), 7.68 (m, 4H), 7.86 (m, 3H), 8.16 (m, 2H), 8.42 (d, *J* = 8 Hz, 1H), 8.48 (d, *J* = 4 Hz, 1H), 8.93 (d, *J* = 8 Hz, 1H).

#### 4.1.2 Synthesis of TPAPV‐NIM‐M

TPAPV‐NIM‐M was synthesized via the Menshutkin reaction. Briefly, 0.1 g (0.159 mmol) of TPAPV‐NIM was treated with iodomethane (in excess) in 2 mL of dimethylformamide (DMF) at 90°C for 12 h. After the reaction was complete, DMF was removed under reduced pressure, and the resulting product was precipitated from ethyl acetate, filtered, and washed with ethyl acetate. The crude product was suspended in acetone (5 mL) without further purification and treated with a saturated solution of potassium hexafluorophosphate (KPF_6_) in deionized water (5 mL) for 2 h at room temperature. Thereafter, acetone was removed under reduced pressure, and the suspension was vacuum‐filtered and washed with water to obtain orange‐red solid (0.086 g, 68.66% yield), which was dried under reduced pressure at 60°C overnight. ^1^H NMR (DMSO‐*d6*, 400 MHz): *δ*/ppm = 2.15 (m, 2H), 3.06 (s, 9H), 3.45 (m, 2H), 4.15 (m, 2H), 7.08 (m, 8H), 7.34 (t, *J* = 8 Hz, 4H), 7.70 (m, 5H), 7.93 (m, 3H), 8.28 (m, 2H), 8.51 (d, *J* = 8 Hz, 1H), 8.56 (d, *J* = 8 Hz, 1H), 9.06 (d, *J* = 8 Hz, 1H). ^13^C NMR (DMSO‐*d6*, 100 MHz): *δ*/ppm = 22.10, 37.31, 52.73, 63.82, 121.03, 123.16, 123.55, 123.90, 124.71, 126.81, 127.56, 127.97, 128.66, 129.48, 130.13, 131.15, 131.41, 133.51, 135.29, 135.61, 140.22, 141.59, 147.38, 164.02, 164.32.

### 4.2 ROS Detection

#### 4.2.1 Singlet Oxygen Detection in Aqueous Medium

Singlet oxygen generation was evaluated using ABDA, a standard commercial indicator for ^1^O_2_ detection. In detail, aqueous solutions (10 µM) of the synthesized PSs (TPA‐NIM‐M, TPAV‐NIM‐M, and TPAPV‐NIM‐M) and ABDA (150 µM) were separately mixed in a 1:1 ratio to obtain 5 µM PS and 75 µM ABDA. The mixtures were irradiated with white light (∼27 mW/cm^2^) for 0–90 s, and the decomposition of ABDA at 400 nm was monitored using a UV–vis spectrophotometer. To assess the effectiveness of the synthesized PSs, the commercial PSs RB (5 µM working solution) and Ce6 (5 µM working solution) were also used, following the same procedure as for synthesized PSs, and the decomposition of ABDA was monitored at 400 nm.

#### 4.2.2 Hydroxyl Radicals (OH•) Detection in Aqueous Medium

Hydroxyl radical (OH^•^) generation was evaluated using hydroxyphenyl fluorescein (HPF), a commercially available standard indicator for OH^•^ detection. Briefly, aqueous solutions (10 µM) of the synthesized PSs (TPA‐NIM‐M, TPAV‐NIM‐M, and TPAPV‐NIM‐M) and HPF (10 µM) were separately mixed in a 1:1 ratio to obtain 5 µM final working solutions of the PSs and HPF. The mixtures were irradiated with white light (∼27 mW/cm^2^) for 0–4 min, and changes in fluorescence intensity at 515 nm were measured with a fluorescence spectrophotometer.

#### 4.2.3 Superoxide Anions (O_2_^•−^) Detection in Aqueous Medium

Superoxide anions (O_2_^•−^) were detected using dihydrorhodamine 123 (DHR‐123), a commercially available indicator for O_2_^•−^ detection. Briefly, aqueous solutions (5 µM) of the PSs (TPA‐NIM‐M, TPAV‐NIM‐M, and TPAPV‐NIM‐M) and DHR‐123 (5 µM) were separately mixed in a 1:1 ratio to obtain 2.5 µM final working solutions of the PSs and DHR‐123. The mixtures were irradiated with white light (∼27 mW/cm^2^) for 0–80 s, and changes in fluorescence intensity at 526 nm were measured using a fluorescence spectrophotometer.

#### 4.2.4 Intracellular ROS Generation

Intracellular ROS generation was evaluated using DCFH‐DA, a commercially available indicator for detecting intracellular ROS. Briefly, MCF‐7 cells were cultured in RPMI‐1640 medium for 24 h, washed with PBS, and treated with TPAPV‐NIM‐M (5 µM) for 2 h. The cells were then washed with PBS, treated with 10 µM DCFH‐DA, incubated for an additional 30 min. After washing with PBS, the cells were irradiated with white light (∼27 mW/cm^2^) for 10 min, and immediately examined using a confocal microscope.

### 4.3 Cytotoxicity Evaluation

#### 4.3.1 Dark Toxicity

MDA‐MB‐231, MCF‐7, 4T1, and A549 cells were separately seeded in 96‐well plates at a density of 5 × 10^3^ cells per well and incubated for 24 h. The cells were then treated with different concentrations (0, 1, 2, 3, 4, and 5 µM) of TPAPV‐NIM‐M and incubated for an additional 24 h. Cell viability was determined using the 3‐(4,5‐dimethyl‐2‐thiazolyl)‐2,5‐diphenyltetrazolium bromide (MTT) assay. Briefly, 10 µL of MTT solution in PBS (5 mg/mL) was added to each well, and the plates were incubated at 37°C for 4 h. The medium containing unreacted MTT was then discarded, and 150 µL of DMSO was added to each well to dissolve the formazan crystals. the plate was shaken for 10 min. Cytotoxicity was evaluated using a multi‐mode plate reader (Biotek, HTX S1 LFA, USA) by measuring absorbance at 570 with 640 nm as the reference wavelength, and cell viability was calculated using the following formula:

$$\begin{array}{cc} \mathrm{Cell}\;\mathrm{viability}\left(\%\right) & =\left[\left(OD_{sample}-OD_{blank}\right)/\left(OD_{control}-OD_{blank}\right)\right]\\ & \;\times 100 \end{array}$$

#### 4.3.2 Light Toxicity

The phototoxicity of TPAPV‐NIM‐M against MDA‐MB‐231, MCF‐7, 4T1, and A549 cells was evaluated using the same procedure employed for the dark toxicity assay, except that white‐light irradiation was used. Briefly, after incubation with TPAPV‐NIM‐M for 2 h, the cells were irradiated with white light (∼27 mW/cm^2^) for 20 min and then incubated for an additional 12 h. Subsequently, MTT solution was added, and the cells were incubated for a further 4 h. Cell viability was determined using the procedure described for the dark toxicity assay.

### 4.4 Live/Dead Cell Staining

MCF‐7 cells (40 × 10^3^ cells) were seeded in a glass‐bottom confocal dish and incubated for 24 h. The cells were then treated with TPAPV‐NIM‐M (5 µM) for 2 h, washed with PBS, and irradiated with white light (∼27 mW/cm^2^) for 10 min. Subsequently, the cells were stained with PI (8 µM) and calcein‐AM (2 µM) and incubated at 37°C for 30 min. The cells were carefully washed with PBS and immediately examined using a confocal microscope as soon as possible. For comparison, MCF‐7 cells (40 × 10^3^) were cultured in a separate glass‐bottom confocal dish as a control group, stained with calcein‐AM/PI for 30 min, then examined using a confocal microscope.

### 4.5 Flow Cytometry Analysis

To assess cellular apoptosis, MCF‐7 and MDA‐MB‐231 cells (1 × 10^6^ cells per cell line) were seeded separately in glass‐bottom confocal dishes and incubated for 24 h. The cells were then treated with TPAPV‐NIM‐M (10 µM), incubated at 37°C for 2 h, and irradiated with white light (∼27 mW/cm^2^) for 12 min. Next, the cells were carefully harvested by trypsinization, then centrifuged at 1500 rpm and 4°C, and washed with PBS. The cells were subsequently stained using an Annexin V‐FITC/PI kit (Thermo Fisher‐Invitrogen) according to the manufacturer's instructions. A total of 10000 cells were collected for flow cytometric analysis. For comparison, MCF‐7 and MDA‐MB‐231 cells (1 × 10^6^ cells) that received neither TPAPV‐NIM‐M treatment nor light irradiation were stained using the same procedure and served as controls.  A total of 10000 cells were collected from each sample for flow cytometric analysis.

### 4.6 Ferroptosis

To assess ferroptosis via confocal microscopy, MCF‐7 and 4T1 cells (60 × 10^3^ cells per cell line) were seeded separately in glass‐bottom confocal dishes and incubated for 24 h. The cells were then treated with TPAPV‐NIM‐M (5 µM) and incubated for 2 h. Subsequently, the cells were washed twice with PBS, stained with C11‐BODIPY‐581/591 (10 µM), incubated for an additional 30 min at 37°C. The cells were then irradiated with light (∼27 mW/cm^2^) for 10 min, washed twice with PBS and imaged using confocal microscopy. For comparison, MCF‐7 and 4T1 cells that received neither TPAPV‐NIM‐M treatment nor light irradiation were stained with C11‐BODIPY‐581/591 (10 µM), incubated for 30 min at 37°C, and imaged using confocal microscopy.

### 4.7 MMP Assay

For the MMP assay, MCF‐7, MDA‐MB‐231, and 4T1 cells (60 × 10^3^ cells per cell line) were seeded separately in glass‐bottom confocal dishes and incubated for 24 h. The cells were then treated with TPAPV‐NIM‐M (5 µM) and incubated for 2 h. Subsequently, the cells were washed with PBS and irradiated with light (∼27 mW/cm^2^) for 5 min. The irradiated cells were then stained with JC‐1 (3 µM), incubated for an additional 30 min at 37°C, washed with PBS, and imaged immediately using confocal microscopy. For comparison, MCF‐7, MDA‐MB‐231, and 4T1 cells (60 × 10^3^ cells per cell line) that received neither TPAPV‐NIM‐M treatment nor light irradiation were stained with JC‐1 (3 µM), incubated for 30 min at 37°C, washed with PBS, and imaged using confocal microscopy.

### 4.8 In Vitro Antibacterial PDT

*S. aureus* and *E*. *coli* suspensions at an OD_600_ of 0.1 were separately incubated with TPAPV‐NIM‐M (0–5 µM) at 37°C for 20 min in the dark. Next, the bacterial suspensions were centrifuged at 8000 rpm for 5 min, washed twice with PBS, and divided into light and dark groups. The light groups were irradiated with white light (∼40 mW/cm^2^) for 20 min and then diluted to 1 × 10^4^ CFU by serial dilution. A 70 µL aliquot of each bacterial suspension (*E*. *coli* or *S*. *aureus*) was spread evenly on an LB‐agar plate and incubated at 37°C for 24 h. For the dark toxicity evaluation, the dark groups were also diluted to 1 × 10^4^ CFU by serial dilution. A 70 µL aliquot of each bacterial suspension (*E*. *coli* or *S*. *aureus*) was pipetted onto an LB‐agar plate, spread evenly over the surface using a cell spreader, and incubated at 37°C for 24 h. To assess the effect of light alone on bacteria, only *S*. *aureus* and *E*. *coli* suspensions (OD_600_ = 0.1) without TPAPV‐NIM‐M treatment were irradiated with white light (∼40 mW/cm^2^) for 20 min and then serially diluted to 1 × 10^4^ CFU. A 70 µL aliquot of each bacterial suspension was pipetted onto an LB‐agar plate, spread evenly over the surface using a cell spreader, and incubated at 37°C for 24 h.

### 4.9 Bacterial Surface Observation

To better understand the antibacterial activity of TPAPV‐NIM‐M, the changes in bacterial morphology were examined using a scanning electron microscope (SEM). To observe differences in bacterial morphology before and after light irradiation, *E*. *coli* and *S*. *aureus* suspensions (OD_600_ = 0.1) were incubated with TPAPV‐NIM‐M (5 µM) for 20 min under shaking conditions, washed with PBS, and irradiated with light (∼40 mW/cm^2^) for 20 min. The bacteria were then deposited on clean 1 × 1 cm^2^ glass slides placed in a 24‐well plate and fixed with 2.5% glutaraldehyde in PBS. The same procedure was used for the dark group, except for the light irradiation. The bacteria were stored at 4°C overnight, dehydrated using a graded ethanol series (30%, 40%, 50%, 60%, 70%, 80%, 90%, and 100%), and examined by SEM to assess morphological changes.

### 4.10 Zeta Potential Study

Zeta potentials of *S*. *aureus* and *E*. *coli* in PBS were measured using a Zetasizer (Malvern). Briefly, *S*. *aureus* and *E*. *coli* (OD_600_ = 0.1) were incubated with TPAPV‐NIM‐M (5 µM) for 20 min, then washed with PBS and analyzed for zeta potential using a Zetasizer. Zeta potentials of untreated *S*. *aureus* and *E*. *coli* in PBS were also measured and used as control groups.

### 4.11 In Vivo Wound Healing Experiment

BALB/c mice (∼6 weeks old) were purchased from the Animal Center at Erciyes University. All animal experiments were conducted in accordance with the approved protocol by the Ethics Committee of Erciyes University (No. 24/216). A skin incision (∼20 mm long) was made on the back of each mouse, and the wound was infected with *E*. *coli* (OD_600_ = 0.1) for 24 h. Thereafter, the mice were randomly divided into four groups (*n* = 3): (1) *E*. *coli* group (only *E*. *coli*), (2) dark group (*E*. *coli* + TPAPV‐NIM‐M), (3) PBS group (*E*. *coli* + PBS + light), and (4) light group (*E*. *coli* + TPAPV‐NIM‐M + light). At 24 h post‐infection with *E*. *coli*, groups (2) and (4) were treated with TPAPV‐NIM‐M (20 µM) and kept at room temperature for 2 h, while group (3) received only PBS treatment. After 2 h, the PBS and light groups were irradiated with white light (∼85 mW/cm^2^) for 25 min, and the wound‐healing progress was monitored photographically.

### 4.12 In Vivo Antitumor PDT

BALB/c female mice (∼6 weeks old) were purchased from the animal center of Erciyes University. The animal experiment was performed in accordance with the approved protocol by the Ethics Committee of Erciyes University (No. 24/216). To establish a tumor model, 4T1 cells (1 × 10^6^ cells) were injected subcutaneously into the right flank of each mouse. After 7 days, when the tumors reached a volume of ∼200 mm^3^, the mice were divided into three groups (PBS, dark, and light). A stock solution of TPAPV‐NIM‐M (10 mM) was prepared in DMSO and diluted to 100 µM in PBS. The PBS group received 100 µL of PBS, while the dark and light groups received intratumoral injections of TPAPV‐NIM‐M (100 µM) in PBS. After 2 h, the light group was irradiated with white light (∼85 mW/cm^2^) for 25 min, and this procedure was repeated on days 1, 3, and 5 during the experiment. The body weights of the mice and tumor volumes were monitored every other day, and the tumor volume was calculated using the following formula:

$$\mathrm{Tumor}\;\mathrm{volume}\left(mm^{3}\right)=\left[length\left(mm\right)\times width^{2}{\left(mm\right)}^{2}\right]/2$$

### 4.13 Blood Hemolysis

To study the hemolytic effect of TPAPV‐NIM‐M, 1 mL of fresh blood was collected from a healthy mouse. The blood was centrifuged at 8000 rpm for 10 min at 4°C to collect the RBCs and washed twice with PBS. RBCs were suspended in 5 mL PBS and vortexed to obtain the RBC suspension. PBS was used as the negative control, and deionized (DI) water as the positive control. To evaluate the hemolytic effect of TPAPV‐NIM‐M, 200 µL of the RBC suspension was added to 800 µL of PBS containing TPAPV‐NIM‐M at a final concentration of 2.5 µM. The same procedure was used to prepare final TPAPV‐NIM‐M concentrations of 5 µM, 10 µM, 20 µM, 30 µM, 50 µM, and 100 µM. For the negative control, 200 µL of RBC suspension was added to 800 µL of PBS, and for the positive control, 200 µL of RBC suspension was added to 800 µL of deionized (DI) water. The samples were then incubated for 3 h at 37°C, and the absorbance was measured using a microplate reader at 577 nm. The hemolysis percentage was calculated using the following formula:

$$\begin{array}{cc} \mathrm{Hemolysis}\left(\%\right) & =\left[\left(A_{sample}-A_{negative\;control}\right)/\right.\\ & \;\left.\left(A_{Positive\;control}-A_{negative\;control}\right)\right]\times 100 \end{array}$$

where *A* represents the absorbance at 577 nm.

### 4.14 H&E Staining

For the infected‐wound model, mice from the *E. coli*, dark, PBS, and light groups were euthanized after 9 days of treatment, and their major organs, including the liver, spleen, heart, lungs, and kidneys, were harvested for H&E staining. For the antitumor PDT experiment, mice from the PBS, TPAPV‐NIM‐M dark, and TPAPV‐NIM‐M + light groups were euthanized at the end of the treatment period, and the tumors and major organs were harvested for H&E staining. The collected tissues were fixed in 10% formaldehyde and stored at 4°C until further processing. For H&E staining, the tissues were dehydrated through a graded ethanol series, cleared with xylene, and embedded in paraffin blocks. Sections with a thickness of 5 µm were obtained using a microtome and stained with H&E. The stained sections were then examined under a light microscope equipped with an LED light source.

## Author Contributions

**Sayed Mir Sayed**: conceptualization, investigation, funding acquisition, writing – original draft, methodology, validation, formal analysis, data curation, project administration. **Zeeshan Tahir**: formal analysis, data curation, writing – original draft. **Fahri Alkan**: formal analysis, validation, software, Writing – original draft. **Yavuz Nuri Ertas**: writing – review and editing, visualization, conceptualization, investigation, funding acquisition, supervision, resources, project administration, validation.

## Funding

This work was supported by the 2232 International Fellowship for the Outstanding Researchers Program of TÜBİTAK (Project No: 121C157). Y.N.E. acknowledges support from the Health Institutes of Türkiye (TÜSEB) Incentive Award, the Turkish Academy of Sciences Distinguished Young Scientist Award (TÜBA‐GEBİP), and the Scientific and Technological Research Council of Türkiye (TÜBİTAK) Incentive Award.

## Conflicts of Interest

The authors declare no conflicts of interest.

## Supporting Information

The Supporting Information is available free of charge and contains additional experimental descriptions, materials and instrumentation, molar absorption coefficients, fluorescence spectra, AIE data, ROS detection via DHR‐123, cytotoxicity, zeta potential, dynamic light scattering data, nanoaggregate stability curves, ferroptosis and apoptosis data, photostability, confocal images of intracellular ROS generation, NMR spectra, high‐resolution mass spectra, real‐time apoptosis tracking, hematological data, and hemolysis evaluation results.

## Supporting information




**Supporting File**: adhm71493‐sup‐0001‐SuppMat.docx.

## Data Availability

The data that support the findings of this study are available from the corresponding author upon reasonable request.
