# Supplementary material for: A Naphthalimide‐Based NIR‐Emissive AIE Photosensitizer for Synergistic Apoptosis/Ferroptosis‐Mediated Antitumor Therapy and Broad‐Spectrum Bacterial Inactivation With Rapid Wound Healing
Source: Adv Healthc Mater. 2026 Jul 29;15(32):e71493. doi: 10.1002/adhm.71493 (PMC13507565; doi:10.1002/adhm.71493)
Supplement: Supplementary file 1 [file ADHM-15-0-s001.docx]

Supporting Information

*for*

**A Naphthalimide-Based NIR-Emissive AIE Photosensitizer for Synergistic**

**Apoptosis/Ferroptosis-Mediated Antitumor Therapy and Broad-Spectrum Bacterial**

**Inactivation with Rapid Wound-Healing**

Sayed Mir Sayed^1^*, Zeeshan Tahir^2^, Fahri Alkan^3^, Yavuz Nuri Ertas^1,4,5,6^*

^1^ ERNAM–Nanotechnology Research and Application Center, Erciyes University, Kayseri, 38039, Türkiye

^2^ Department of Medical Biochemistry, Faculty of Medicine, Erciyes University, Kayseri, Türkiye

^3^ Department of Chemistry, Bilkent University, Ankara 06800, Türkiye

^4^ Department of Biomedical Engineering, Erciyes University, Kayseri 38039, Türkiye

^5^ ERFARMA Drug Application and Research Center, Erciyes University, 38039, Kayseri, Türkiye

^6^ UNAM–Institute of Materials Science and Nanotechnology, Bilkent University, Ankara 06800, Türkiye

Corresponding Authors:

Sayed Mir Sayed, ERNAM–Nanotechnology Research and Application Center, Erciyes University, Kayseri, 38039, Türkiye, E-mail: sayed.mir.sayed5@gmail.com

Yavuz Nuri Ertas, Department of Biomedical Engineering, Erciyes University, Kayseri 38039, Türkiye, E-mail: yavuznuri@gmail.com

**Materials and Instrumentation**

4-(Diphenylamino)phenyl boronic acid, 4-bromostyrene, 4-bromo-1,8-naphthalic anhydride, tetrakis(triphenylphosphine) palladium (0) (Pd(PPh_3_)_4_), palladium (II) acetate, and sodium carbonate were purchased from Ambeed. 3-Dimethylaminopropylamine was purchased from Tokyo Chemical Industry. Triethylamine, iodomethane, potassium hexafluorophosphate, and DCFH-DA were purchased from Sigma-Aldrich. Calcein-AM/PI and Annexin V-FITC/PI kits were purchased from Thermo Fisher Scientific. Wheat Germ Agglutinin (WGA) Conjugates were purchased from Biotium, DiO and Hoechst 33342 were purchased from MedChemExpress, and Anthracenediyl-bis-(methylene)-dimalonic acid (ABDA) was obtained from BLDpharm. Hydroxyphenyl fluorescein (HPF), dihydrorhodamine 123 (DHR-123), Z-VAD-FMK (Z-VAD), and Ferrostatin-1 (Fer-1) were purchased from Targetmol. Organic solvents were purchased from the local commercial suppliers and used as received without further purification. Nuclear magnetic resonance (NMR) spectroscopy (*via* a Bruker DRX-400 MHz spectrometer) was used to characterize the chemical structures of the synthesized molecules in CDCl_3_ or DMSO-*d6* at room temperature. UV−vis and fluorescence spectra were recorded *via* a UV−vis spectrophotometer (Perkin Elmer Lambda-25) and a spectrofluorophotometer (Cary Eclipse, Germany). Zeta potentials of *S*. *aureus* and *E*. *coli* were measured on a Zetasizer (Malvern Nano ZS-90), and the surface morphologies of bacteria were measured via a scanning electron microscope (SEM) (ZEISS GEMINI-500). A confocal laser scanning microscope (ZEISS LSM-900, 63x /oil confocal mode) was used to record the confocal images. Cell apoptosis was assessed by flow cytometry (FACSAria III), and data were analyzed using FlowJo version 10.0.7. Hematoxylin and Eosin (H&E)–stained tissue sections/slices were observed under a light microscope (ZEISS Axio Imager 2 Upright Microscope–Imager.Z2m) using an LED light source.

**Cell Culture**

**Mammalian Cells**

MDA-MB-231, Saos-2, HUVECs, and 4T1 cells were cultured in Dulbecco’s modified Eagle’s medium (DMEM) supplemented with 10% fetal bovine serum (FBS) and 1% penicillin−streptomycin at 37 °C in a humid atmosphere (5% CO_2_). MCF-7 breast cancer cells were cultured in Roswell Park Memorial Institute medium (RPMI) 1640 cell culture medium supplemented with 10% fetal bovine serum (FBS) and 1% penicillin−streptomycin at 37 °C in a humid atmosphere (5% CO_2_). A549 lung cancer cells were cultured in Kaighn's Modification of Ham's F-12 Medium (F12K) cell culture medium supplemented with 10% fetal bovine serum (FBS) and 1% penicillin−streptomycin at 37 °C in a humid atmosphere (5% CO_2_).

**Ferroptosis/Apoptosis Detection**

Ferroptosis/apoptosis was detected using Fer-1 (5 μM) or (Z-VAD) (10 μM), which were incubated separately with 4T1 cells (60×10^3^ cells) for 2 h in separate glass-bottomed confocal dishes, followed by treatment with TPAPV-NIM-M (5 μM) and light (⁓27 mW/cm^2^) for 5 min. After that, the cells were washed with PBS and observed under a confocal microscope. The same procedure was adopted for the control groups, except for incubation with TPAPV-NIM-M. For the MTT assay, 5×10^3^ cells were used in each well of 96-well plates and treated with Fer-1 (20 μM), Z-VAD (40 μM), and TPAPV-NIM-M (0-5 μM) for 2 h. Depending upon the nature of the experiment, the experimental groups were subjected to white-light irradiation (⁓27 mW/cm^2^) for 5 min, incubated for a further 12 h, and cell viability was measured by MTT assay.

**Bacterial Cells**

20 µL of *S. aureus* and 20 µL of *E. coli* suspensions from -20 °C bacterial stock were separately added to 5 mL of freshly prepared Luria–Bertani broth (LB-broth) and incubated at 37 °C for 12 h under shaking conditions (200 RPM). Thereafter, the bacteria were harvested by centrifugation at 8000 RPM for 5 min; the supernatant was discarded, and the bacteria were washed with PBS and used for further experiments.

**Laser Scanning Confocal Fluorescence Imaging**

To evaluate the imaging performance of TPAPV-NIM-M, live cells were treated with 5 μM TPAPV-NIM-M at 37 °C for 30 min. Thereafter, the cells were washed with PBS, treated with 2 μM Hoechst 33342, and incubated at 37 °C for 5 min. After 5 min of incubation, the cells were washed twice with PBS and observed under a confocal microscope with a 63× oil-immersion objective. Likewise, the subcellular distribution of TPAPV-NIM-M was studied using the same procedure described above, in which cells were incubated with TPAPV-NIM-M (5 μM) and WGA-conjugates (5 μM) or DiO (10 μM) for 30 min. After PBS washing, the stained cells were treated with 2 μM Hoechst 33342 and incubated for 5 min, then observed under a confocal laser scanning microscope with a 63× oil-immersion objective.

Next, to evaluate the photostability, MCF-7 breast cancer cells were stained with TPAPV-NIM-M as described above. Next, the fluorescence-labeled cells were continuously irradiated with the confocal microscope's laser for 5 min at 10% of its full output, and confocal images were acquired every 20 s during irradiation.

To perform the bacterial imaging experiments, Staphylococcus aureus (*S*. *aureus*) and Escherichia coli (*E*. *coli*) (OD_600_ = 0.1) were separately incubated with TPAPV-NIM-M (5 µM) at 37 °C under shaking (200 RPM) for 20 min. Next, the bacteria were centrifuged at 8000 RPM for 5 min, washed with PBS, and treated with DAPI (20 µM) for 10 min at 37 ^o^C under shaking conditions (200 RPM), followed by PBS washing. For confocal imaging, bacteria were resuspended in PBS, and 20.0 µL of TPAPV-NIM-M and DAPI-stained bacteria were dropped on the glass slide, covered with a microscope coverslip, and observed under a confocal laser scanning microscope with a 63× oil-immersion objective. The confocal fluorescence images were acquired under the excitation of a 488 nm laser for TPAPV-NIM-M and 405 nm for DAPI.

**Computational Details**

All computations were performed using the ADF 2023 package [1]. The electronic structure calculations were carried out employing the B3LYP [2, 3] exchange–correlation functional in combination with the DZP (double-ζ polarized) basis set. Geometry optimizations were performed for both the ground and first excited states. Solvent effects were incorporated using the COSMO implicit solvation model. Spin–orbit coupling (SOC) matrix elements were evaluated within the perturbative framework based on the ZORA (Zero-Order Regular Approximation) Hamiltonian [4].

**Dynamic Light Scattering Measurements**

Zeta potential and size of the nanoaggregates were measured *via* dynamic light scattering (DLS) techniques. To measure the zeta potential of TPAPV-NIM-M, a 1 mM solution of TPAPV-NIM-M in DMSO was diluted to 5 µM in deionized water, and the zeta potential was measured *via* DLS. For the size of nanoaggregates of TPAPV-NIM-M, a 1 mM solution of TPAPV-NIM-M in DMSO was diluted to 5 µM in PBS, and the size of nanoaggregates was measured by DLS.

**Synthetic Procedures**

**Synthesis of DAP-NIM and TPA-NIM**

DAP-NIM and TPA-NIM were synthesized according to our previously reported protocol [5].

**Synthesis of TPA-PV**

TPA-PV was synthesized by a Suzuki cross-coupling reaction between 4-(diphenylamino)phenylboronic acid (TPA-B(OH)_2_) and 4-bromostyrene. Briefly, 0.6 g (3.278 mmol) 4-bromostyrene, 1.043 g (3.606 mmol) TPA-B(OH)_2_, 0.095 g Pd(PPh_3_)_4_ (0.082 mmol), and 1.042 g (9.833 mmol) sodium carbonate were added to a Schlenk flask; the flask was sealed, evacuated, and backfilled with nitrogen. Subsequently, 21 mL of toluene, 6 mL of ethanol, and 3 mL of deionized water were added. The mixture was purged with nitrogen and then refluxed at 90 °C for 12 h under nitrogen. After completion of the reaction, as confirmed by thin-layer chromatography (TLC), the reaction mixture was washed with deionized water and brine, dried with anhydrous sodium sulfate, and concentrated under reduced pressure. The impure product was purified by column chromatography on silica gel using a dichloromethane/n-hexane (1:6) mixture as the eluent, affording a white solid (yield: 80.95%). ^1^H-NMR (CDCl_3_, 400 MHz): δ/ppm = 5.27 (d, *J* = 12 Hz, 1H), 5.80 (d, *J* = 20 Hz, 1H), 6.77 (m, 1H), 7.05 (t, *J* = 8 Hz, 2H), 7.16 (m, 6H), 7.29 (m, 4H), 7.49 (m, 4H), 7.56 (d, *J* = 8 Hz, 2H). ^13^C-NMR (CDCl_3_, 100 MHz): δ/ppm = 113.60, 122.97, 123.85, 124.45, 126.67, 127.57, 129.29, 134.56, 136.11, 136.45, 140.02, 147.25, 147.65. **Synthesis of TPA-V**

TPA-V was synthesized *via* the Wittig reaction. Briefly, 0.5g of 4-(diphenylamino)benzaldehyde (1.830 mmol) and 0.784g of methyltriphenylphosphonium bromide (2.195 mmol) were taken in anhydrous tetrahydrofuran (THF) (10 mL) in a round-bottom flask; the mixture was cooled to 0 °C and stirred at 0 °C for 15 min. Thereafter, 0.308g potassium tert-butoxide (2.745 mmol) was dissolved in 5 mL of anhydrous THF and added dropwise to the stirring mixture at 0 °C; the resulting mixture was stirred at 25 °C overnight. After reaction completion, as confirmed by TLC, the reaction mixture was concentrated under reduced pressure, and the resulting oily residue was dissolved in DCM, extracted with brine, dried over anhydrous sodium sulfate, and concentrated under reduced pressure. The impure product was purified by column chromatography on silica gel using n-hexane as the eluent to obtain 0.327g of a colorless, oily product, which was dried under vacuum at 50 °C overnight to yield a white solid (yield: 65.85%). ^1^H-NMR (CDCl_3_, 400 MHz): δ/ppm = 5.20 (m, 1H), 5.69 (m, 1H), 6.72 (m, 1H), 7.11 (m, 7H), 7.33 (m, 7H).

**Synthesis of TPA-NIM-M**

TPA-NIM-M was synthesized *via* the Menshutkin reaction. Briefly, 0.150 g (0.285 mmol) of TPA-NIM was treated with iodomethane (in excess) in 3 mL of dimethylformamide (DMF) at 90°C for 12 h. After the reaction was complete, DMF was removed under reduced pressure, and the resulting product was precipitated from ethyl acetate, filtered, and washed with ethyl acetate. It was taken in 5 mL of acetone without further purification and treated with a saturated solution of potassium hexafluorophosphate (KPF_6_) in deionized water (5 mL) for 2 h at room temperature. Thereafter, the acetone was removed under reduced pressure, and the suspension was vacuum-filtered and washed with water to obtain 0.126 g of orange-red solid (yield: 64.48%), which was dried under reduced pressure at 60°C overnight. ^1^H-NMR (DMSO-*d6*, 400 MHz): δ/ppm = 2.16 (m, 2H), 3.03 (s, 9H), 3.44 (m, 2H), 4.17 (m, 2H), 7.14 (m, 8H), 7.38 (t, *J* = 8 Hz, 4H), 7.47 (d, *J* = 8 Hz, 2H), 7.81 (d, *J* = 8 Hz, 1H), 7.88 (t, *J* = 8 Hz, 1H), 8.40 (d, *J* = 8 Hz, 1H), 8.54 (t, *J* = 8 Hz, 2H). ^13^C-NMR (DMSO-*d6*, 100 MHz): δ/ppm = 22.09, 37.14, 52.63, 63.62, 121.32, 122.40, 122.98, 124.32, 125.27, 127.87, 129.63, 130.25, 130.98, 131.29, 131.46, 131.74, 146.37, 147.23, 148.19, 164.00, 164.29.

**Synthesis of TPAV-NIM**

TPAV-NIM was synthesized *via* the Heck cross-coupling reaction. Briefly, 0.250 g (0.692 mmol) of DAP-NIM, 0.188 g (0.692 mmol) TPA-V, 0.016 g (0.0692 mmol) palladium (II) acetate, and 0.042 g (0.138 mmol) tri(o-tolyl) phosphine were taken in a Schlenk flask, and the flask was degassed multiple times. 20 mL of dry acetonitrile and 1.350 mL of triethylamine were injected, respectively. The mixture was purged with nitrogen and refluxed at 90°C for 48 h under nitrogen. Thereafter, acetonitrile was removed under reduced pressure, and the resulting reaction mixture was taken up in dichloromethane (DCM), extracted with deionized water, washed with brine, dried over anhydrous sodium sulfate, filtered, and concentrated on a rotary evaporator. The resulting impure product was purified by column chromatography on silica gel using a 1:2 methanol/tetrahydrofuran mixture, affording 0.192 g of a reddish solid (yield: 50.29%). ^1^H-NMR (DMSO-*d6*, 400 MHz): δ/ppm = 1.75 (m, 2H), 2.13 (s, 6H), 2.30 (m, 2H), 4.06 (t, *J* = 8 Hz, 2H), 6.97 (d, *J* = 8 Hz, 2H), 7.08 (m, 6H), 7.34 (t, *J* = 8 Hz, 4H), 7.48 (d, *J* = 16 Hz, 1H), 7.72 (d, *J* = 8 Hz, 2H), 7.83 (m, 1H), 7.98 (d, *J* = 16 Hz, 1H), 8.13 (d, *J* = 8 Hz, 1H), 8.45 (m, 2H), 8.86 (m,1H).

**Synthesis of TPAV-NIM-M**

TPAV-NIM-M was synthesized *via* the Menshutkin reaction. Briefly, 0.125 g (0.226 mmol) of TPAV-NIM was treated with iodomethane (in excess) in 2 mL of dimethylformamide (DMF) at 90°C for 12 h. After the reaction was complete, DMF was removed under reduced pressure, and the resulting product was precipitated from ethyl acetate, filtered, and washed with ethyl acetate. It was taken in 5 mL of acetone without further purification and treated with a saturated solution of potassium hexafluorophosphate (KPF_6_) in deionized water (5 mL) for 2 h at room temperature. Thereafter, the acetone was removed under reduced pressure, and the suspension was vacuum-filtered and washed with water to obtain 0.109 g of dark-red solid (yield: 67.77%), which was dried under reduced pressure at 60 °C overnight. ^1^H-NMR (DMSO-*d6*, 400 MHz): δ/ppm = 2.18 (m, 2H), 3.06 (s, 9H), 4.17 (m, 2H), 7.07 (m, 8H), 7.39 (m, 4H), 7.58 (m, 1H), 7.77 (m, 2H), 7.91 (m, 1H), 8.06 (m, 1H) , 8.22 (m, 1H), 8.52 (m, 2H), 8.97 (s, 1H). ^13^C-NMR (DMSO-*d6*, 100 MHz): δ/ppm = 22.15, 37.06, 52.67, 63.62, 120.64, 121.49, 122.55, 122.84, 123.42, 124.23, 125.08, 127.35, 128.70, 129.30, 129.41, 130.17, 130.84, 131.05, 131.08, 131.32, 135.25, 141.91, 147.17, 148.36, 163.92, 164.27.

**Scheme S1.** Schematic illustration of the synthesis of TPA-NIM-M, TPAV-NIM-M, and TPAPV-NIM-M.

**Figure S1.** Molar extinction coefficient (ɛ) of TPAPV-NIM-M, TPAV-NIM-M, and TPA-NIM-M in DMSO. Concentration: 20 µM.

**Figure S2.** a) Fluorescence emission spectra of TPA-NIM-M in chloroform. b) Fluorescence emission spectra of TPA-NIM-M in DMSO/toluene mixtures with different toluene fractions based on volume. c) Plot of the changes in the fluorescence intensities of TPA-NIM-M with varying fractions of toluene. d) Fluorescence emission spectra of TPAV-NIM-M in chloroform. e) Fluorescence emission spectra of TPAV-NIM-M in DMSO/toluene mixtures with different toluene fractions based on volume. f) Plot of the changes in the fluorescence intensities of TPAV-NIM-M with varying fractions of toluene.

**Figure S3.** a) Degradation of ABDA (75 µM) in the presence of Ce6 (5 µM) under white light irradiation (⁓27 mW/cm^2^). b) Degradation of ABDA (75 µM) in the presence of RB (5 µM) under white light irradiation (⁓27 mW/cm^2^).

**Figure S4.** Changes in the fluorescence emission spectra of DHR-123 (2.5 µM) under white light irradiation (⁓27 mW/cm^2^) in the presence of a) TPA-NIM-M, b) TPAV-NIM-M, and c) TPAPV-NIM-M. d) Relative fluorescence intensity curves of DHR-123 in the presence of TPA-NIM-M, TPAV-NIM-M, and TPAPV-NIM-M. Concentrations of TPA-NIM-M, TPAV-NIM-M, and TPAPV-NIM-M were 2.5 µM.

**Figure S5.** Theoretical absorption spectra for the TPAPV-NIM-M molecule in chloroform and water, calculated with the B3LYP/DZP level of theory and the COSMO solvent model.

**Figure S6.** Cell viability of (a) HUVECs and (b) MDA-MB-231 cells treated with TPAPV-NIM-M (0–40 µM) in the dark.

**Figure S7.** Photostability of TPAPV-NIM-M (5 µM) in the MCF-7 cell line under 10% laser power for 5 min of continuous irradiation. Images were captured every 20 s of laser irradiation. The arrows indicate a slight increase in cell swelling after 5 min of laser irradiation. Scale bar: 10 µm.

**Figure S8.** Detailed time-lapse confocal fluorescence images of TPAPV-NIM-M (5 µM)-treated MCF-7 cells during the second laser-irradiation period, corresponding to cumulative irradiation times of 3–6 min. Images were captured every 20 s under continuous laser scanning at 30% laser power. The sequence shows the onset of PM blebbing. Scale bar: 10 µm.

**Figure S9.** Continuation of the time-lapse sequence shown in Figure S8. Confocal fluorescence images of TPAPV-NIM-M (5 µM)-treated MCF-7 cells during the third laser-irradiation period, corresponding to cumulative irradiation times of 6–9 min. Images were captured every 20 s under continuous laser scanning at 40% laser power. The sequence shows progressive formation of more numerous and larger PM blebs. Scale bar: 10 µm.

**Figure S10.** Hemolysis results of the red blood cells treated with different concentrations of TPAPV-NIM-M.

**Figure S11.** ^1^H NMR spectrum of TPA-PV.

**Figure S12.** ^13^C NMR spectrum of TPA-PV.

**Figure S13.** ^1^H NMR spectrum of TPA-V.

**Figure S14.** ^1^H NMR spectrum of TPA-NIM.

**Figure S15.** ^1^H NMR spectrum of TPA-NIM-M. Note: Signals from the two methylene protons in the upfield region partially overlapped with signals from water as impurities in the NMR solvent (DMSO-*d6*). It can be further confirmed from the ^13^C NMR spectrum of TPA-NIM-M (**Figure S34**).

**Figure S16.** ^13^C NMR spectrum of TPA-NIM-M.

**Figure S17.** High-resolution mass spectrum (HRMS) of TPA-NIM-M.

**Figure S18.** ^1^H-NMR spectrum of TPAV-NIM.

**Figure S19.** ^1^H-NMR spectrum of TPAV-NIM-M. Note: Signals from the two methylene protons in the upfield region fully overlapped with the signals from water as impurities in the NMR solvent (DMSO-*d6*). It can be confirmed from the ^13^C NMR spectrum of TPAV-NIM-M (**Figure S38**).

**Figure S20.** ^13^C NMR spectrum of TPAV-NIM-M.

**Figure S21.** High-resolution mass spectrum (HRMS) of TPAV-NIM-M.

**Figure S22.** ^1^H NMR spectrum of TPAPV-NIM.

**Figure S23.** ^1^H NMR spectrum of TPAPV-NIM-M.

**Figure S24.** ^13^C NMR spectrum of TPAPV-NIM-M.

**Figure S25.** High-resolution mass spectrum (HRMS) of TPAPV-NIM-M.

| **Table S1.** Singlet and triplet excited state energies, oscillator strengths, configurations, and Λ indexes for the S_0_ geometry of TPAPV-NIM-M calculated with the B3LYP/DZP level of theory and chloroform as solvent. | | | | | |
| --- | --- | --- | --- | --- | --- |
| Excited State | Energy  (eV) | *f*  (a.u.) | Transition | Weight | Λ index |
| Singlet Excited States | | | | |  |
| 1 | 2.25 | 0.53 | H→L | 0.97 | 0.25 |
| 2 | 2.83 | 0.67 | H-1→L | 0.96 | 0.68 |
| 3 | 3.21 | 0.63 | H→L+1 | 0.94 | 0.46 |
| 4 | 3.61 | 0.03 | H-2→L | 0.93 | 0.68 |
| 5 | 3.77 | 0.05 | H→L+4 | 0.97 | 0.59 |
| Triplet Excited States | | | | |  |
| 1 | 1.98 | - | H-1→L | 0.49 | 0.53 |
| 2 | 2.35 | - | H→L | 0.52 | 0.46 |
| 3 | 2.75 | - | H-2→L | 0.37 | 0.58 |
| 4 | 3.01 | - | H→L+1 | 0.28 | 0.57 |
| 5 | 3.31 | - | H→L+5 | 0.74 | 0.60 |

| **Table S2.** Singlet and triplet excited state energies, oscillator strengths, configurations, and Λ indexes for the S_1_ geometry of TPAPV-NIM-M calculated with the B3LYP/DZP level of theory and chloroform as solvent. | | | | | |
| --- | --- | --- | --- | --- | --- |
| Excited State | Energy  (eV) | *f*  (a.u.) | Transition | Weight | Λ index |
| Singlet Excited States | | | | |  |
| 1 | 1.83 | 0.92 | H→L | 0.97 | 0.34 |
| 2 | 2.48 | 0.61 | H-1→L | 0.97 | 0.72 |
| 3 | 2.96 | 0.65 | H→L+1 | 0.95 | 0.53 |
| 4 | 3.31 | 0.01 | H-2→L | 0.94 | 0.69 |
| 5 | 3.61 | 0.12 | H-5→L | 0.34 | 0.59 |
| Triplet Excited States | | | | |  |
| 1 | 1.51 | - | H-1→L | 0.52 | 0.54 |
| 2 | 1.99 | - | H→L | 0.52 | 0.36 |
| 3 | 2.51 | - | H-2→L | 0.37 | 0.58 |
| 4 | 2.74 | - | H→L+1 | 0.28 | 0.57 |
| 5 | 3.15 | - | H→L+5 | 0.74 | 0.60 |

| **Table S3.** Singlet and triplet excited state energies, oscillator strengths, configurations, and Λ indexes for the S_0_ geometry of TPAPV-NIM-M calculated with the B3LYP/DZP level of theory and water as solvent. | | | | | |
| --- | --- | --- | --- | --- | --- |
| Excited State | Energy  (eV) | *f*  (a.u.) | Transition | Weight | Λ index |
| Singlet Excited States | | | | |  |
| 1 | 2.25 | 0.52 | H→L | 0.97 | 0.25 |
| 2 | 2.82 | 0.68 | H-1→L | 0.96 | 0.68 |
| 3 | 3.20 | 0.62 | H→L+1 | 0.94 | 0.46 |
| 4 | 3.61 | 0.03 | H-2→L | 0.94 | 0.68 |
| 5 | 3.77 | 0.04 | H→L+4 | 0.97 | 0.60 |
| Triplet Excited States | | | | |  |
| 1 | 1.98 | - | H-1→L | 0.49 | 0.53 |
| 2 | 2.35 | - | H→L | 0.53 | 0.47 |
| 3 | 2.74 | - | H-2→L | 0.37 | 0.58 |
| 4 | 3.01 | - | H→L+1 | 0.28 | 0.57 |
| 5 | 3.31 | - | H→L+5 | 0.77 | 0.57 |

| **Table S4.** Singlet and triplet excited state energies, oscillator strengths, configurations, and Λ indexes for the S_1_ geometry of TPAPV-NIM-M calculated with the B3LYP/DZP level of theory and water as solvent. | | | | | |
| --- | --- | --- | --- | --- | --- |
| Excited State | Energy  (eV) | *f*  (a.u.) | Transition | Weight | Λ index |
| Singlet Excited States | | | | |  |
| 1 | 1.76 | 1.22 | H→L | 0.97 | 0.42 |
| 2 | 2.48 | 0.44 | H-1→L | 0.97 | 0.73 |
| 3 | 2.90 | 0.69 | H→L+1 | 0.96 | 0.58 |
| 4 | 3.29 | 0.01 | H-2→L | 0.94 | 0.70 |
| 5 | 3.58 | 0.04 | H-5→L | 0.35 | 0.62 |
| Triplet Excited States | | | | |  |
| 1 | 1.41 | - | H→L | 0.62 | 0.55 |
| 2 | 1.98 | - | H-1→L | 0.54 | 0.64 |
| 3 | 2.44 | - | H→L+1 | 0.58 | 0.61 |
| 4 | 2.71 | - | H-2→L | 0.37 | 0.65 |
| 5 | 3.15 | - | H-5→L | 0.83 | 0.60 |

| **Table S5.** Calculated SOC matrix elements (cm^-1^) between singlet and triplet excited states for the S_0_ geometry of TPAPV-NIM-M calculated with the B3LYP/DZP level of theory and water as solvent. | | | | | |
| --- | --- | --- | --- | --- | --- |
| States | T_1_ | T_2_ | T_3_ | T_4_ | T_5_ |
| S_1_ | 0.32 | 0.19 | 0.78 | 0.19 | 0.32 |
| S_2_ | 0.23 | 0.50 | 0.58 | 0.62 | 0.06 |
| S_3_ | 0.38 | 0.36 | 0.38 | 0.38 | 1.18 |
| S_4_ | 0.27 | 0.62 | 0.41 | 0.32 | 0.02 |
| S_5_ | 0.35 | 0.51 | 0.55 | 0.93 | 1.11 |

| **Table S6.** Calculated SOC matrix elements (cm^-1^) between singlet and triplet excited states for the S_1_ geometry of TPAPV-NIM-M calculated with the B3LYP/DZP level of theory and water as solvent. | | | | | |
| --- | --- | --- | --- | --- | --- |
| States | T_1_ | T_2_ | T_3_ | T_4_ | T_5_ |
| S_1_ | 0.21 | 0.16 | 0.59 | 0.09 | 0.15 |
| S_2_ | 0.24 | 0.30 | 0.20 | 0.27 | 0.24 |
| S_3_ | 0.41 | 0.18 | 0.25 | 0.15 | 0.02 |
| S_4_ | 0.33 | 0.57 | 0.21 | 0.23 | 0.31 |
| S_5_ | 0.30 | 0.43 | 0.50 | 0.37 | 0.17 |

**References**

[1] G.T. Te Velde, F.M. Bickelhaupt, E.J. Baerends, C. Fonseca Guerra, S.J. van Gisbergen, J.G. Snijders, T. Ziegler, Chemistry with ADF, Journal of Computational Chemistry, 22 (2001) 931-967.

[2] A.D. Becke, Density‐functional thermochemistry. III. The role of exact exchange, The Journal of Chemical Physics, 98 (1993) 5648-5652.

[3] C. Lee, W. Yang, R.G. Parr, Development of the Colle-Salvetti correlation-energy formula into a functional of the electron density, Physical Review B, 37 (1988) 785-789.

[4] E. van Lenthe, J.G. Snijders, E.J. Baerends, The zero‐order regular approximation for relativistic effects: The effect of spin–orbit coupling in closed shell molecules, The Journal of Chemical Physics, 105 (1996) 6505-6516.

[5] S.M. Sayed, K.-F. Xu, H.-R. Jia, F.-F. Yin, L. Ma, X. Zhang, A. Khan, Q. Ma, F.-G. Wu, X. Lu, Naphthalimide-based multifunctional AIEgens: Selective, fast, and wash-free fluorescence tracking and identification of Gram-positive bacteria, Analytica Chimica Acta, 1146 (2021) 41-52.
